# Mosaic divergent repeat interruptions of motif length and sequence, mDRILS, of XDP-linked repeats affect formation of DNA/RNA triplexes & quadruplexes

**DOI:** 10.1371/journal.pone.0360550

**Published:** 2026-10-27

**Authors:** Niousha Sharifi Soltani, Bita Zamiri, Amirreza Shafieeha, Ali Akbar Moosavi-Movahedi, Joshua Laß, Christine Klein, Ana Westenberger, Joanne Trinh, Sebastian Hönes, Christopher E. Pearson

**Affiliations:** 1 Department of Chemical Engineering, Amirkabir University of Technology (Tehran Polytechnic), Iran; 2 Institute of Biochemistry and Biophysics (IBB), University of Tehran, Tehran, Iran; 3 Institute of Neurogenetics, University of Lübeck, Lübeck, Germany; 4 Department of Endocrinology, Diabetes and Metabolism, University Hospital Essen, University of Duisburg-Essen, Essen, Germany; 5 Department of Molecular and Medical Genetics, University of Toronto, Canada; 6 The Hospital for Sick Children, Toronto, Canada; NMIMS Deemed to be University - Mumbai Campus: NMIMS, INDIA

## Abstract

An inserted SINE-VNTR-Alu(AGAGGG)n repeat into the *TAF1* gene is the cause of X-linked dystonia-parkinsonism. *M*osaic *d*ivergent *r*epeat *i*nterruptions affecting both motif *l*ength and *s*equence (mDRILS) of the canonical repeat (AGAGGG)n arise and can diminish repeat instability and delay disease age of onset. The repeat itself may affect gain-of-function disease pathways. Consequently, XDP etiology may involve unusual structures formed by the repeats. To study the effects of mDRILS on the formation of unusual structures of the XDP *TAF1*-SVA repeat, we designed a series of G-rich DNA and RNA oligonucleotides having the pure canonical repeat 5’-(AGAGGG)4−3’, or either of the most common mDRILS 5’-(AGAGGG)2***AGGG***(AGAGGG)-3’; and 5’-(AGAGGG)2***AGG***(AGAGGG)-3’. Circular dichroism, thermal melting, binding of the G-quadruplex ligand TMPyP4, shows the first canonical and ***AGGG***-interrupted sequence assume K^+^-sensitive stable G-quadruplexes, whereas GA duplexes are the prominent structure in the ***AGG***-interrupted form. The complementary C-rich strands, 5’-(CCCTCT)4−3’, and 5’-(CCCTCT)2***CCCT***(CCCTCT)-3’, and 5’-(CCCTCT)2***CCT***(CCCTCT)-3’, assumed *i*-motif-compatible structure. Mixtures of the G- and C-rich complementary sequences, at 1:2 ratios, showed triplex-forming propensity for the canonical and AGGG-interrupted, but not the AGG-interrupted forms. The ability of mDRILS to elicit modifications of the disease-causing XDP *TAF1*-SVA repeat to enable or disable the formation of specific structures, such as quadruplex and triplex structures, may explain in part how mDRILS mediate their disease-modifying effects.

## Introduction

X-linked dystonia-parkinsonism (XDP) is a severe neurodegenerative adult-onset movement disorder endemic to individuals of Filipino descent, clinically characterized by progressive dystonia and parkinsonism [1,2]. XDP is caused by a SINE-VNTR-Alu (SVA)-type retrotransposon insertion with a variable tandem repeat, d(AGAGGG)n on the anti-sense strand in intron 32 of the *TAF1* gene, encoding the TATA-binding protein-associated factor 1. This *TAF1* SVA insertion is a founder mutation unique to Filipinos from the island of Panay. The length of the d(AGAGGG)n repeat of the *TAF1* SVA varies between patients, and longer repeat tracts have been associated with earlier disease age at onset (AAO) [3], similar to other repeat expansion disorders [1]. The length of the inherited *TAF1* SVA repeat may be further exacerbated by ongoing somatic expansion instability of repeat lengths, which affects both AAO and pathogenic characteristics’ tissue specificity [4]. Somatic expansions are evident in the basal ganglia and cerebellum [5,6]. Understanding how the SVA insertion and the repeat expansions lead to disease is critical for diagnosis, prognosis, and therapeutic advances.

XDP is associated with both gain-of-function (GOF) and loss-of-function (LOF) etiologies [1]. For example, in cell models, splicing of the *TAF1* transcript is disrupted by the SVA insertion and results in a decrease in the full-length transcript levels [7,8]. Inherited mutations in *TAF1*, in the absence of the SVA insertion, are causative for neurodevelopmental phenotypes, cardiac anomalies, and cancer [9]. Heterozygous and homozygous losses of *TAF1* in mice do not appear to lead to XDP-like phenotypes in male or female mice [10]. Targeted mutations result in morphological and functional issues with the cerebellum and cerebral cortex of mice [11]. Downregulation of the TAF1 protein or its function is expected to dysregulate the expression of many genes, as TAF1 is a key subunit of the TFIID transcription complex. The SVA insertion, likely through the ability of the repeat to form a quadruplex structure, has been linked to reduced *TAF1* transcription, aberrant *TAF1* splicing, and reduced TAF1 protein [12,13]. Alternate pathogenic paths are that XDP is due to an SVA-mediated GOF, including non-canonical repeat-associated non-AUG translation [12] and transcriptional protein sequestration via binding to the expanded repeats. The formation of unusual DNA/RNA structures by the expanded repeats are suspected to block *TAF1* transcription, alter *TAF1* splicing, act in RNA foci, RNA-binding protein sequestration, RAN-translation, and repeat instability. Thus, understanding factors that affect the ability of the SVA repeat to adopt unusual structures is warranted.

Sequence variations that interrupt the purity of the *TAF1* SVA canonical repeat d(AGAGGG)n tract, as well as variations that change the length of the repeating motif, are known to arise and have biological effects upon repeat instability and disease etiology [14,15]. Specifically, *m*osaic *d*ivergent *r*epeat *i*nterruption affecting motif *l*ength and *s*equence, termed mDRILS, were identified in the *TAF1* SVA, and these mDRILS affect repeat stability in XDP and AAO [14,15]. Various mDRILS have been identified within the canonical repeat d(AGAGGG), and their frequency has been shown to be associated with increased stability of the XDP repeat and to delay AAO. The two most prominent mDRILS are ***AGGG*** units [5’-SINE-VNTR-Alu(AGAGGG)2***AGGG***(AGAGGG)n] and ***AGG*** units [5’-SINE-VNTR-Alu(AGAGGG)2***AGG***(AGAGGG)n] [14,15]. *In silico* predictions suggest that the d(AGAGGG)n repeat-containing SVA domains, can assemble into unusual non-B-DNA structures, specifically four-stranded G-quadruplexes (G4-DNA), with the repeat region showing the highest potential [3]. Indeed, some evidence supports a role for quadruplex formation by the XDP repeat [16,17]. However, the consequence of mDRILS on the structural organization of the repeat has not been understood and it is unclear whether these sequence interruptions simply modify the stability of the structures formed or remodel the structural landscape by altering the relative propensity for different secondary structure formation. In addition, d(AGAGGG)n, being comprised of only adenines and guanines, both purines, while the complementary strand d(CCCTCT)n is comprised solely of cytosines and thymines, both imidines. Triplex DNA (H-DNA) or three-stranded DNA formation occurs in sequences enriched with homopurine-homopyrimidine mirror repeats. Therefore, the XDP-linked *TAF1*-SVA repeat has been suggested to hypothetically form triplex DNA [18] yet triplex formation by these repeats has not been addressed experimentally.

DNA and RNA sequences rich in guanine can form higher-order structures through the formation of a cyclic planar array, known as a G-quartet, comprised of eight Hoogsteen hydrogen bonds between four guanine molecules. G-quartets stack on top of each other self-assembling into inter- or intra-molecular four-stranded structures known as G-quadruplexes (Fig 1A) [19,20]. Their formation is critically dependent on the coordination of stabilizing metal cations such as potassium (K^+^) and sodium (Na^+^) within the central cavity of the G-quartets. G-quadruplexes are extremely polymorphic structures. Environmental factors such as cation type and concentration, pH, temperature, flanking nucleotides, and loop sequences play crucial roles in the overall stability of G-quadruplexes. When guanine-rich strands are present, complementary strands are naturally cytosine-rich. Cytosine-rich strands can also form non-B-DNA four-stranded structures known as intercalated motif, or *i-*motifs under appropriate conditions (Fig 1B) [21]. A slightly acidic pH stabilizes *i*-motif structures, which allows for the formation of C − C^+^ base pairs [22,23]. Finally, triplex-stranded DNA structures are formed when a third strand of DNA binds to the major groove of the double-stranded DNA (Fig 1C). Formed typically from homopurine/homopyrimidine sequences, these structures contain Hoogtseen or reverse Hoogsteen hydrogen bonds via the third strand in addition to Watson-Crick hydrogen bonds in the double-stranded DNA. Subsequently, C•G-*C*^*+*^ and T•A-*T* triads form in pyrimidine-motif DNA triplexes where cytosine needs to be protonated in the former (the third strand nucleotide is italicized). Hence, these triplex DNA structures are also usually stabilized under acidic environmental conditions as the protonation of cytosine bases in the third strand is crucial for triplex stability [24,25].

**Fig 1:**
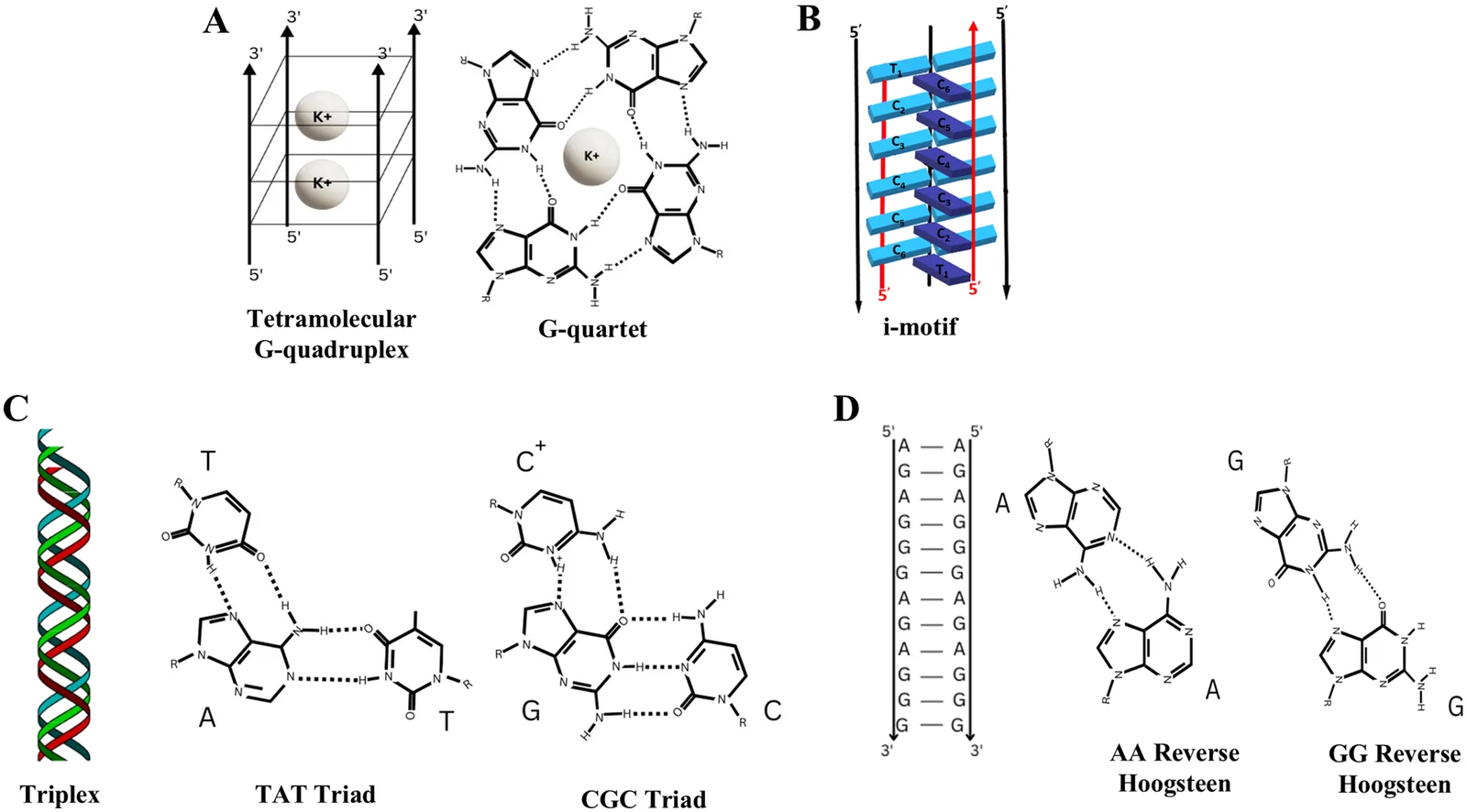
(A) A tetramolecular G-quadruplex and a planar G-quartet (B) An *i-*motif (C) A triple-2stranded DNA and the base triads found in triplexes (D) A parallel GA duplex and the typical base pairs found in them.

The molecular mechanism by which the *TAF1*-SVA repeat (AGAGGG)•(CCCTCT) and/or its mDRILS influence XDP are yet to be fully understood. G-rich and C-rich repeat tracts are, in isolation, known to form G-quadruplexes and *i-*motifs, respectively, and together can form triplex DNAs in presence of one strand in excess, yet the role of sequence variations, as seen in mDRILS in XDP-linked repeats, on the ability to form non-B DNA structures are poorly understood. Deciphering key aspects of the nucleic acid structures is an important step in elucidating the disease etiology. Here we have assessed the formation of non-B DNA structures by G-rich DNA/RNA and C-rich DNA oligonucleotides with XDP-linked repeats based on the canonical repeat and the two most prominent mDRILS (Table 1) both individually and in mixtures of complementary strands.

**Table 1:** Oligonucleotides used in this study.

Name	Oligonucleotide (5’ to 3’)	Molar Extinction Coefficient ($\varepsilon (\frac{L}{mol.cm})$	G to A ratio
**XDP-dG1**	AGA GGG AGA GGG AGA GGG AGA GGG	313760.00	2
**XDP-dG2**	AGA GGG AGA GGG **AGGG** AGA GGG	286550.00	2.143
**XDP-dG3**	AGA GGG AGA GGG **AGG** AGAGGG	274540.00	2
**XDP-dC1**	CCC TCT CCC TCT CCC TCT CCC TCT	180000.00	NA
**XDP-dC2**	CCC TCT **CCCT** CCC TCT CCC TCT	164550.00	NA
**XDP-dC3**	CCC TCT **CCT** CCC TCT CCC TCT	157500.00	NA
**XDP-rG3**	rArGrA rGrGrG rArGrA rGrGrG r**ArGrG r**ArGrArGrGrG	235500.00	2

## Results

### Sequence-dependent quadruplex formation by G-rich DNA/RNA of XDP repeats

Circular dichroism spectroscopy (CD spectroscopy) is an analytical technique used to gain information about the folding patterns of biomolecules including nucleic acids. Based on the structures formed by DNA or RNA oligonucleotides, signature CD peaks are observed. To study the potential effects of mDRILS on structure formation relative to the canonical XDP *TAF1*-SVA repeat, we designed a series of G-rich oligonucleotides with four repeat units each, where the first is the pure canonical repeat; XDP-dG1 = [5’-(AGAGGG)4−3’]; and the next two contain the most common mDRILS, XDP-dG2 = [5’-(AGAGGG)2***AGGG***(AGAGGG)-3’]; and XDP-dG3 = [5’-(AGAGGG)2***AGG***(AGAGGG)-3’].

We assessed the folding patterns of these oligos and the effect of cation type (K^+^ versus Li^+^) using CD spectroscopy (Fig 2). All three G-rich sequences displayed positive peaks at 260 nm and 210 nm, a negative peak at 240 nm and a shoulder at 220 nm, all of which are signature peaks associated with parallel G-quadruplexes. A unique characteristic of G-quadruplexes is that their formation depends on the type of cation present. While sodium (Na^+^) and potassium (K^+^) ions are known to stabilize G-quadruplexes, lithium (Li^+^) tends to disrupt their folding [26,27]. Lithium ions can destabilize G-quadruplexes due to their small ionic radius and low affinity for the O6 atoms of the guanines in the G-quartets. Therefore, comparison of folding patterns in the presence of the two different cations can be indicative of the presence of G-quadruplexes [27]. As seen from Fig 2, Li^+^ does not significantly affect the CD spectra of either of the three G-rich DNA sequences as the signature peaks are conserved. The minimal effect of cation type on CD spectra, as seen in Fig 2, has been previously reported for GA-rich sequences where GA duplexes are present in solution as they also exhibit signature peaks around 265, 250, and 215 nm [28–30]. An example of a parallel GA duplex with G-G and A-A pairs is shown in Fig 1D. However, for XDP-dG2, it is seen that the 220 nm peaks increased in intensity in Li^+^. Also, XDP-dG2 has higher intensity peaks in comparison to the two other sequences with the same concentration (Fig 2A). As an orthogonal approach to address these CD-detected structural differences, we performed thermal melting experiments.

**Fig 2:**
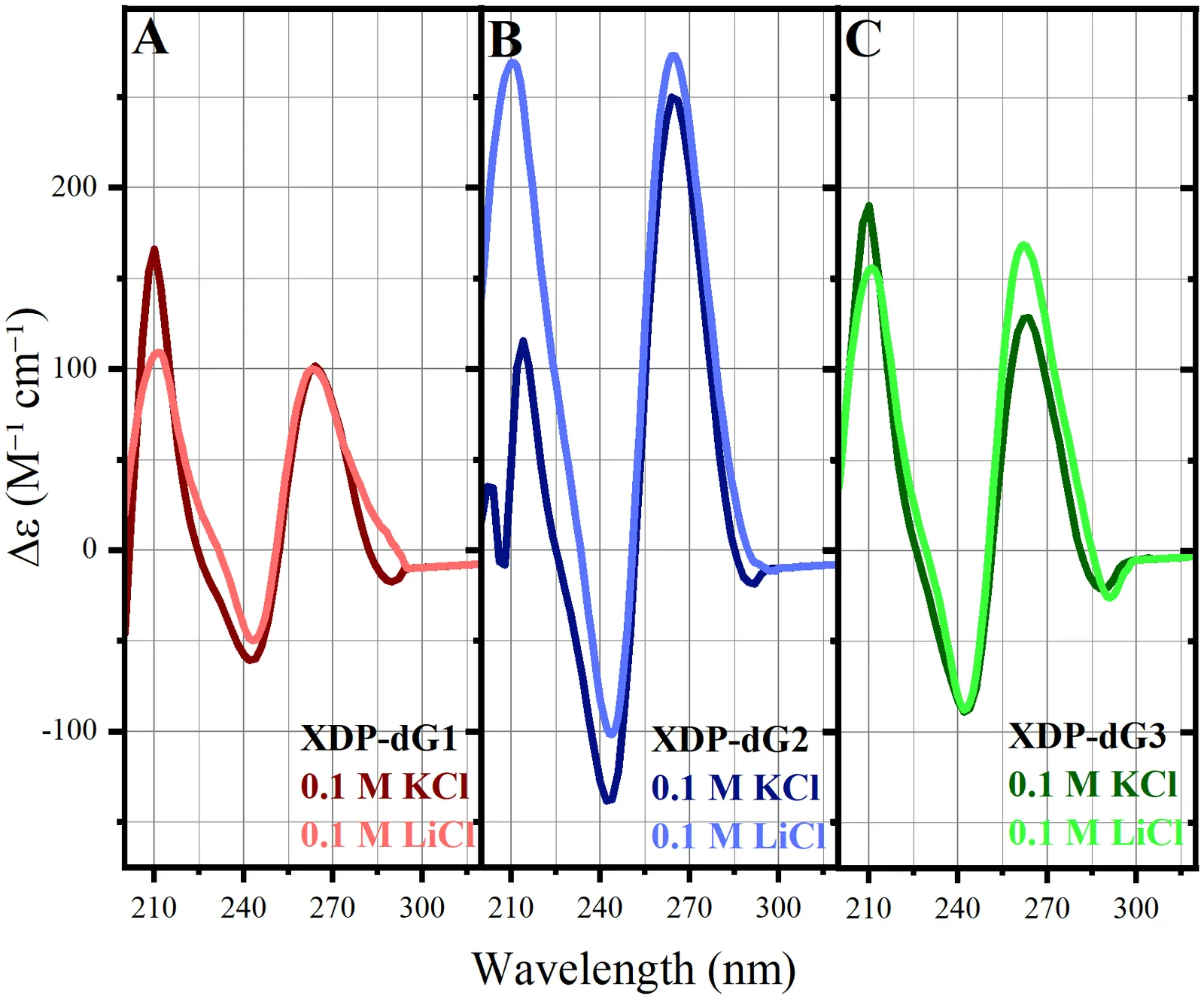
CD spectra of (A) XDP-dG1, (B) XDP-dG2, and (C) XDP-dG3 were recorded using 5 μM oligonucleotide in 10 mM Tris-HCl and 0.1 mM EDTA at pH 7.4, containing either 100 mM KCl or 100 mM LiCl. The dark-colored and light-colored curves represent the KCl and LiCl conditions, respectively. XDP-dG1 = [5’-(AGAGGG)4−3’], XDP-dG2 = [5’-(AGAGGG)2***AGGG***(AGAGGG)-3’]; and XDP-dG3 = [5’-(AGAGGG)2***AGG***(AGAGGG)-3.

Ultraviolet melting curves, obtained by monitoring UV absorbance versus temperature, are used to measure the thermal properties such as melting temperature (T_m_) and to provide information about multiple folding patterns exhibited by each repeat. UV thermal melting experiments were performed for all G-rich DNA repeats in K^+^ and Li^+^ (Fig 3). For all three repeats, melting patterns depended on the type of cation present. In K^+^, two thermal transitions are evident for both XDP-dG1 (T_m_ = 33.69 ± 0.25 °C and 61.79 ± 0.4 °C) and XDP-dG2 (T_m_ = 35.23± 0.66°C and 68.12± 0.9 °C). However, in LiCl, only one transition is observed for XDP-dG1 (T_m_ = 46.45 ± 0.22 °C) and XDP-dG2 (T_m_ = 45.70 ± 0.08 °C). For XDP-dG3, one prominent melting transition was observed in both KCl and LiCl. For all three sequences, in Li^+^, the melting temperatures were about 9°C higher than the lower Tm in K^+^ and the melting transition exhibited less noise, showing a more thermally stable structure in Li^+^ (Fig 3, compare panels A and B with D and E). This is consistent with previous studies that show Li^+^ stabilizes GA duplexes [28]. Comparing the melting transitions in K^+^ versus Li^+^, K^+^ results in a high degree of structural polymorphism as the melting transitions exhibit more noise, and also, in XDP-dG1 and XDP-dG2, two prominent transitions were seen, indicating the presence of thermally distinguishable conformational populations. Since K^+^ stabilizes G-quadruplexes and Li^+^ does not, higher melting temperatures seen for XDP-dG1 and XDP-dG2, further confirm possible G-quadruplex formation as they are known to exhibit higher thermal stability relative to other unusual nucleic acid folding patterns. Among the three sequences studied, XDP-dG2 exhibits folding patterns with the highest melting temperatures. The presence of predominantly one melting transition for XDP-dG3 may be indicative of a lower tendency towards G-quadruplex formation. The presence of two melting transitions for XDP-dG1 and XDP-dG2 was confirmed by calculating the derivative of absorbance versus temperature (S1 Fig.) [27,31]. The former suggests the presence of multiple stable structures, showing a high degree of polymorphism. Interestingly, S1 Fig. also highlights the proximity of the first melting transition for all three sequences in KCl. To further confirm the presence of two folding patterns, including G-quadruplexes by XDP-dG2 and XDP-dG1, thermal difference spectra (TDS) was recorded for all three G-rich sequences in K^+^ or Li^+^.

**Fig 3:**
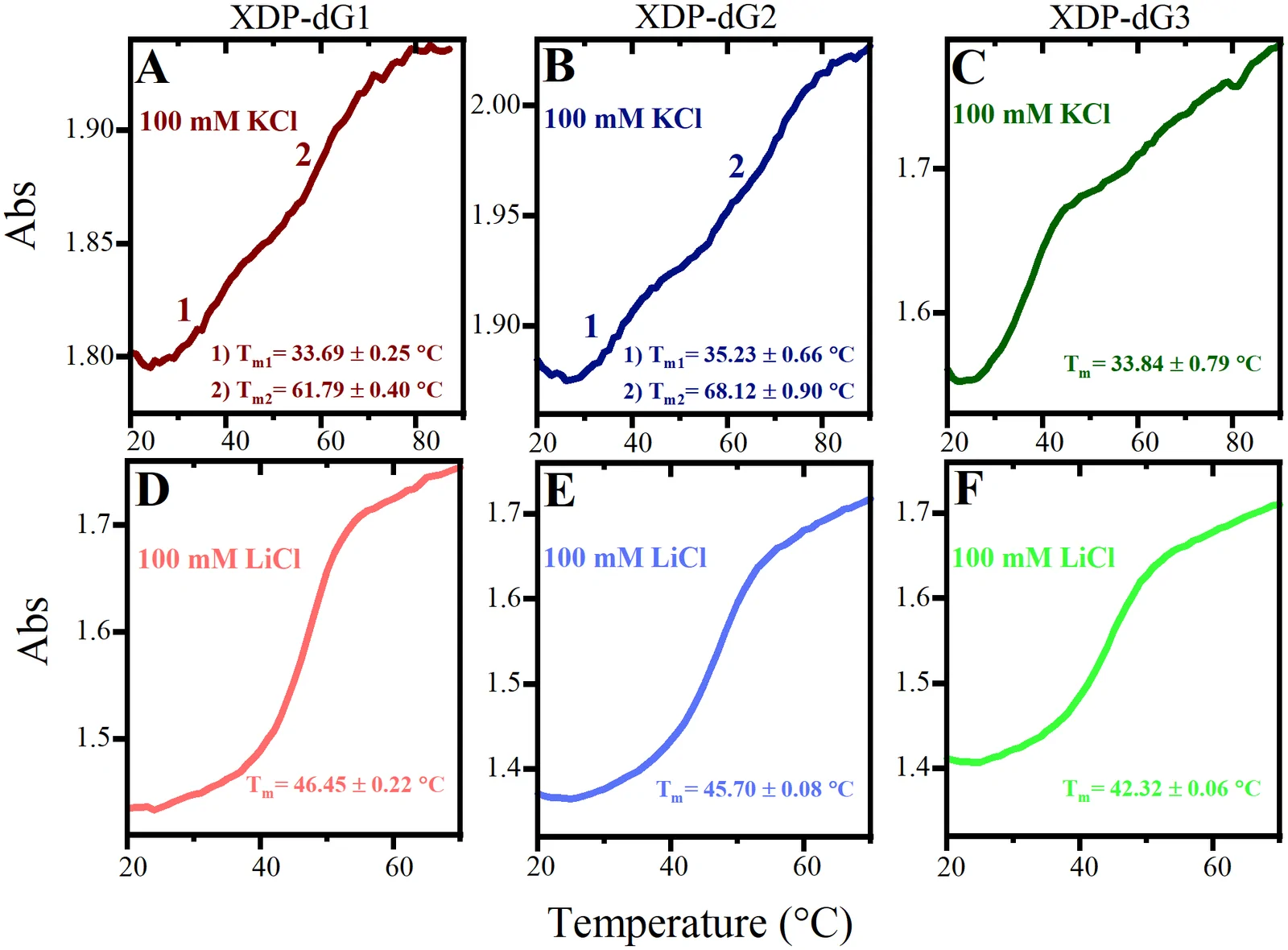
Thermal melting profiles of (A) XDP-dG1, (B) XDP-dG2, and (C) XDP-dG3 were recorded at 260 nm using 5 μM oligonucleotide in 10 mM Tris-HCl and 0.1 mM EDTA at pH 7.4, containing either 100 mM KCl (upper panels; 20 to 90 °C) or 100 mM LiCl (lower panels; 20 to 70 °C). XDP-dG1 = [5’-(AGAGGG)4−3’], XDP-dG2 = [5’-(AGAGGG)2***AGGG***(AGAGGG)-3’]; and XDP-dG3 = [5’-(AGAGGG)2***AGG***(AGAGGG)-3’].

Thermal difference spectra (TDS) are the difference in the UV spectra of folded versus unfolded structures and provides a signature spectra for different folding patterns and can be used as a complementary method to differentiate between duplex DNA, G-quadruplexes, triplexes and etc. For instance, positive peaks at 243 nm and 273 nm alongside a prominent 295 peak in the TDS spectra have been associated with G-quadruplex formation [32]. The TDS for all three G-rich sequences in K^+^ or Li^+^ are shown in Fig 4. The effect of cation type was pronounced for XDP-dG1 and XDP-dG2, where two distinct spectra arose depending upon cation type for each sequence. In K^+^, both sequences exhibit a sharp negative peak at 295 nm, a signature peak for G-quadruplex formation where this negative peak disappeared in Li^+^ (Fig 4A and 4C). However, for XDP-dG3, the TDS for K^+^ and Li^+^ were nearly superimposed showing minimum dependence on cation type. TDS peaks at 258 nm alongside a minor 293 nm peak as shown in the TDS spectra of XDP-dG3 have been associated with GA duplexes [32]. Notably, the TDS spectra of XDP-dG1 and XDP-dG2 in Li^+^ and XDP-dG3 in both K^+^ and Li^+^ have similar spectral features. Therefore, the TDS results are consistent with the melting experiment results, which confirm the tendency of XDP-dG1 and XDP-dG2 to form G-quadruplexes in K^+^. The distinct structure formed by the various XDP repeat motifs may suggest differential propensities to interact with ligands. Previous studies have shown that G + A-rich sequences can form homoduplexes in Li ⁺ -containing conditions and tetraplexes in K ⁺ -containing conditions, with the G-quadruplex population displaying greater thermal stability [33]. Based on this literature and the combined thermal-melting, CD, ion-dependence, and TDS results, the lower- and higher-temperature transitions observed in K⁺ were tentatively assigned to GA-homoduplex and G-quadruplex, respectively.

**Fig 4:**
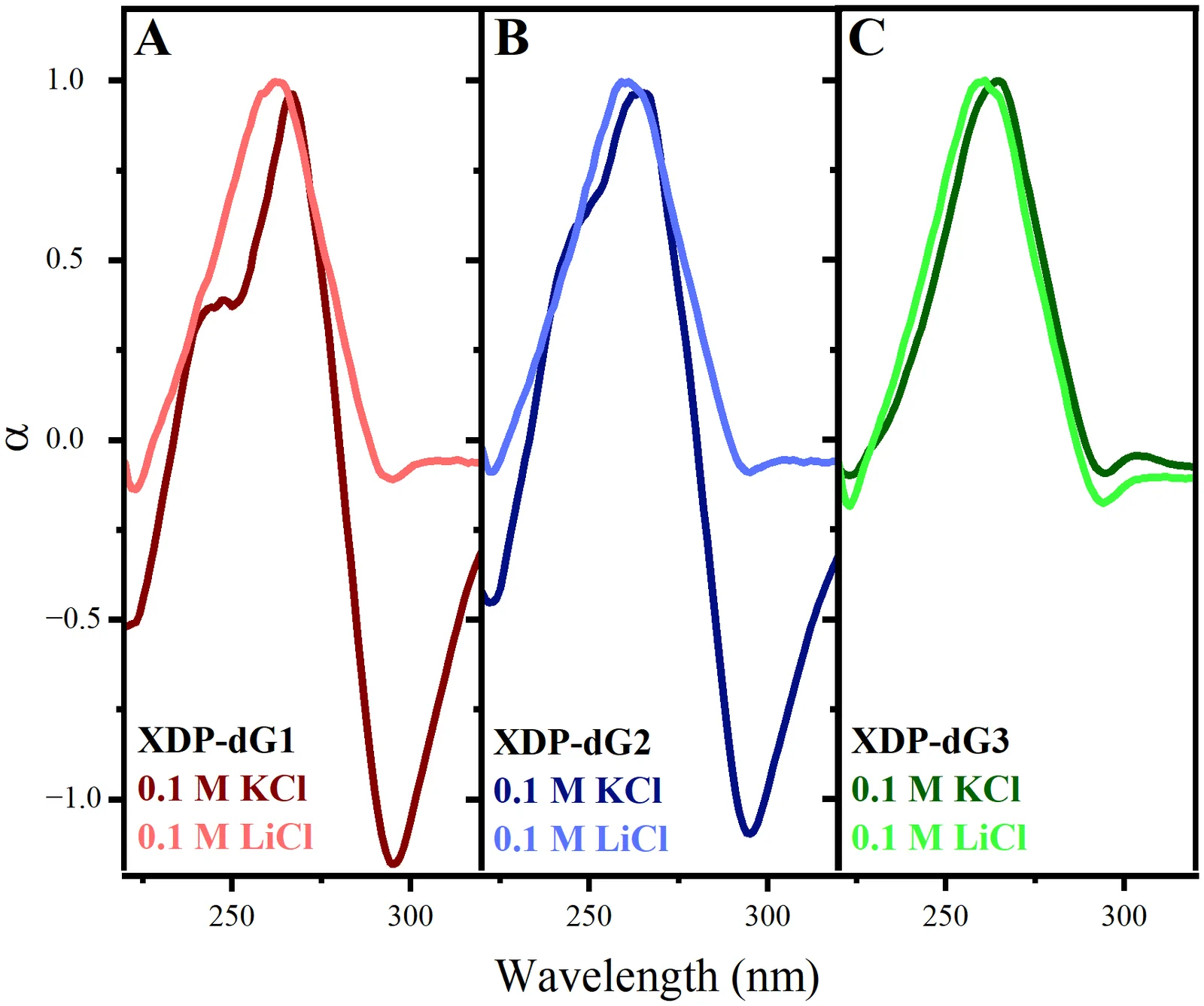
Normalized thermal difference spectra of (A) XDP-dG1, (B) XDP-dG2, and (C) XDP-dG3 between 220−340 nm wavelength, using 5 μM oligonucleotide concentration in 10 mM Tris-HCl and 0.1 mM EDTA at pH 7.4, containing either 100 mM KCl or 100 mM LiCl. The dark-colored and light-colored curves represent the KCl- and LiCl-containing conditions, respectively. XDP-dG1 = [5’-(AGAGGG)4−3’], XDP-dG2 = [5’-(AGAGGG)2***AGGG***(AGAGGG)-3’]; and XDP-dG3 = [5’-(AGAGGG)2***AGG***(AGAGGG)-3’].

The porphyrin TMPyP4 has a high affinity for G-quadruplex nucleic acid structures [34]. The interaction between TMPyP4 and the structures formed by XDP-dG1, XDP-dG2 and XDP-dG3 were studied using the shift in the Soret band upon increasing the DNA concentration (Fig 5). The binding curves can be seen in the inset of Fig 5. The hypochromicity recorded (decrease of absorbance upon binding) was 65.5%, 82.2%, and 69.3% for XDP-dG1, XDP-dG2 and XDP-dG3, respectively, suggesting interaction between TMPyP4 and all sequences with preferential binding to XDP-dG2 due to the highest level of hypochromicity. XDP-dG1 and XDP-dG2 exhibited a 21 nm bathochromic or red shift upon binding whereas XDP-dG3 exhibited a 20 nm red shift, reflective of the change in the electronic states of the ligand when bound to DNA. The lack of an isosbestic point for XDP-dG2 (Fig 5B) suggests that TMPyP4 binding does not simply follow a two-state model. Therefore, singular value decomposition (SVD) analysis was performed on the XDP-dG2 titration data (S3-S4 Figs and S1 Table) [35–37]. The results indicate a multi-step model rather than a simple two-state model for TMPyP4 binding to XDP-dG2. TMPyP4 exhibited stronger binding to XDP-dG1 compared to XDP-dG3 (K_b_ = 1.82 × 10^6^ M^-1^ versus 1.21 × 10^6^ M^-1^). Alongside the data from the previous sections, the differential TMPyP4 binding affinity and the difference in the binding paths for different XDP-linked DNA repeats reflects the difference in folding patterns present and may point towards higher G-quadruplex populations in XDP-dG2.

**Fig 5:**
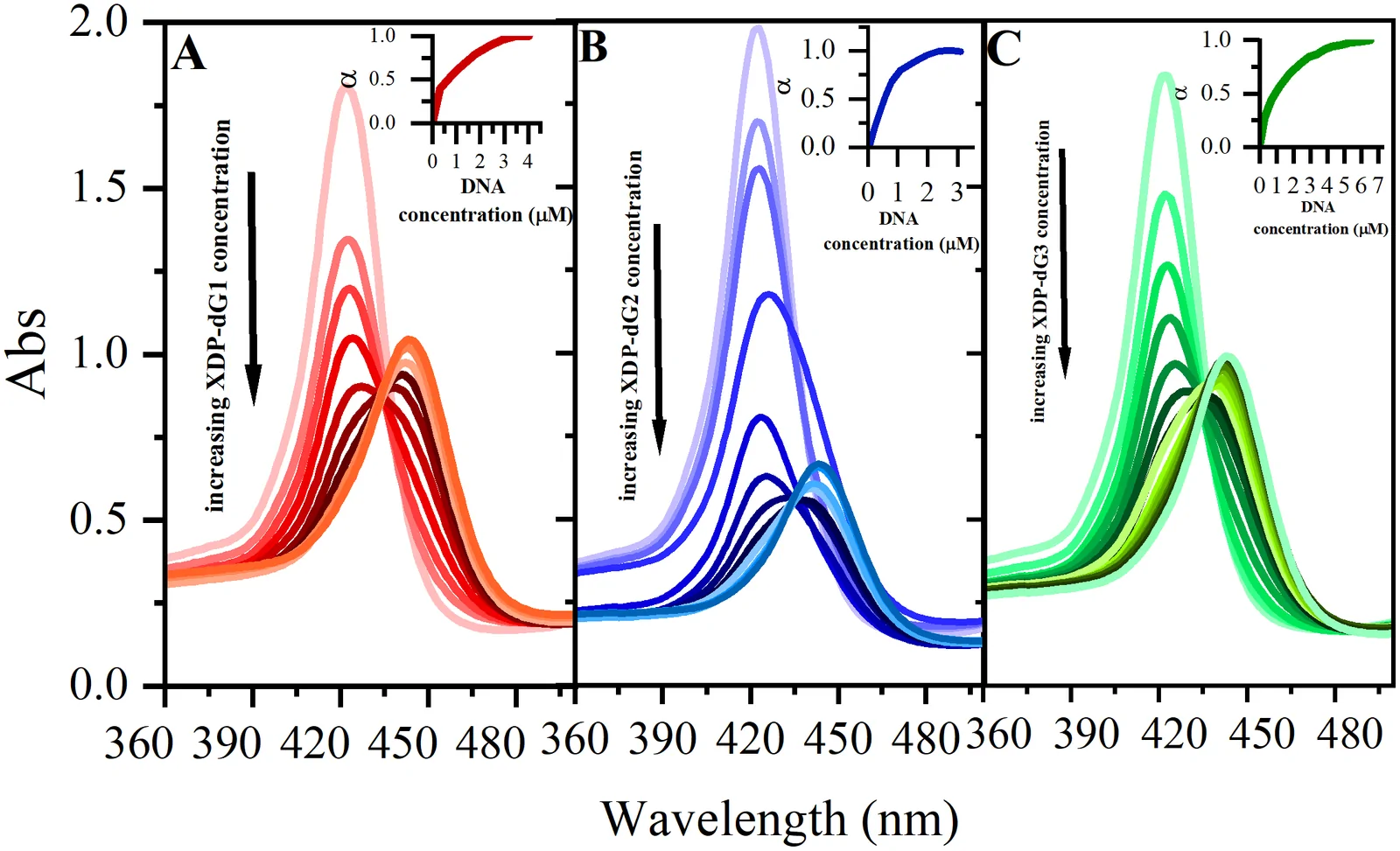
Soret-band shifts observed during TMPyP4 binding to (A) XDP-dG1, (B) XDP-dG2, and (C) XDP-dG3. Titrations were performed using 7 μM TMPyP4 in 10 mM Tris-HCl, 0.1 mM EDTA, and 100 mM KCl at pH 7.4. Each panel shows absorption spectra recorded following successive additions of the corresponding G-rich oligonucleotide. The oligonucleotide titrants were prepared in the same buffer to maintain consistent buffer composition and ionic strength throughout the titrations. XDP-dG1 = [5’-(AGAGGG)4−3’], XDP-dG2 = [5’-(AGAGGG)2***AGGG***(AGAGGG)-3’]; and XDP-dG3 = [5’-(AGAGGG)2***AGG***(AGAGGG)-3’].

### Sequence-dependent G-quadruplex formation by G-rich RNA of XDP repeats

To study the potential effects of mDRILS on structure formation in the repeat of the *TAF1*-SVA RNA transcript, the structures assumed by the mDRILS with an AGG insert, XDP-rG3 = [5’-r(AGAGGG)2***AGG***(AGAGGG)-3’] were studied (Fig 6). Considering that the mDRILS sequence with an AGG insertion was shown to have minimal G-quadruplex formation within XDP-dG3, the DNA sequence, possible formation of G-quadruplexes in XDP-rG3 was studied in detail. This is because RNA G-quadruplexes tend to be more stable than DNA G-quadruplexes with the same sequence, which is due to the presence of the 2’-hydroxyl groups in RNA, which enhances hydrogen bonding and overall structural stability [38]. The CD spectra of XDP-dG3 exhibit positive peaks at 265 nm and 295 nm and a negative peak at 220 nm. In Li^+^, the 295 nm peak is absent and the 220 nm peak has significant reduction of intensity. The CD spectra of XDP-rG3 has atypical characteristics. Specifically, XDP-rG3 seems to be forming a mixture of parallel and antiparallel G-quadruplexes. The considerable effect of Li^+^ ions on the CD spectra (Fig 6A), the observation of a melting transition is recorded for XDP-rG3 at 295 nm (Fig 6B), a signature wavelength for melting G-quadruplexes, and the TDS spectra (Fig 6C) all point towards G-quadruplex formation in XDP-rG3. Although mostly RNA G-quadruplexes have been reported to be parallel-stranded, antiparallel RNA G-quadruplexes have been observed before as well [39]. However, since only XDP-rG3 was examined at the RNA level, this observation cannot be generalized to other XDP mDRILS. Next, the structures assumed by the complementary cytosine-rich DNA strands were studied.

**Fig 6:**
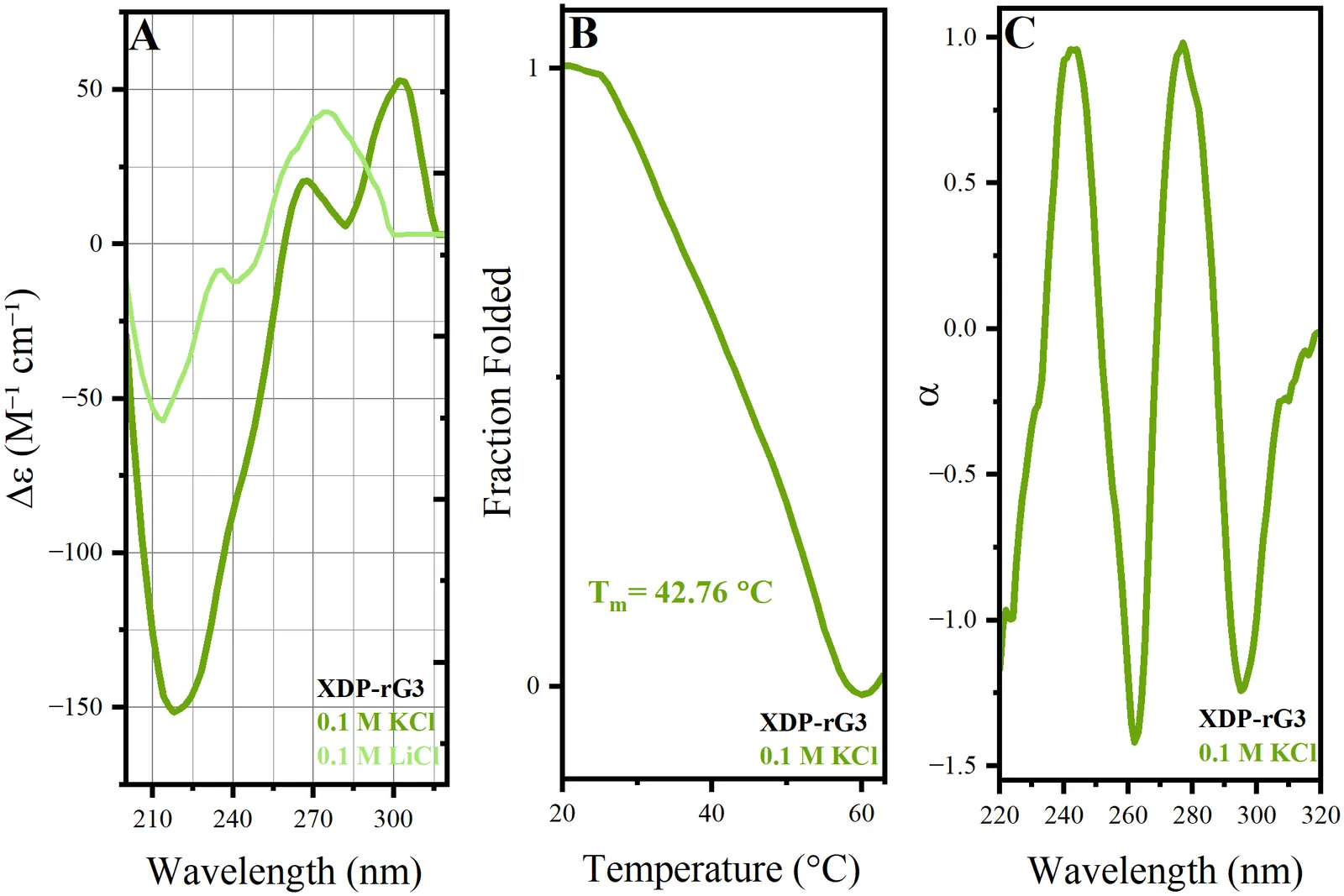
Structural characterization of XDP-rG3 in 10 mM Tris-HCl and 0.1 mM EDTA at pH 7.4, containing either 100 mM KCl or 100 mM LiCl: (A) CD spectra, (B) thermal melting profiles recorded at 295 nm, and (C) normalized thermal difference spectra. The KCl- and LiCl-containing conditions are indicated by dark green and light green, respectively. XDP-rG3 = [5’-(rArGrArGrGrG)2***rArGrG***(rArGrArGrGrG)-3’].

### Sequence-dependent i-motif-compatible structure formation by C-rich DNA of XDP repeats

C-rich sequences can adopt *i-*motif structures under mildly acidic conditions, where protonation of cytosine at the N3 position facilitates the formation of hemiprotonated C·C⁺ base pairs [40,41]. To study the effects of mDRILS on structure formation in the C-rich strands under slightly acidic environment, we have designed a series of C-rich oligonucleotides with four repeat units each, where the first is the pure canonical repeat; XDP-dC1 = [5’-(CCCTCT)4−3’]; and the next two contain the most common mDRILS, XDP-dC2 = [5’-(CCCTCT)2***CCCT***(CCCTCT)-3’]; and XDP-dC3 = [5’-(CCCTCT)2***CCT***(CCCTCT)-3’] (Fig 1F). Using CD spectroscopy, TDS spectra and UV melting transitions, the secondary structures formed by XDP-dC1, XDP-dC2 and XDP-dC3 have been studied (Fig 7). All three C-rich sequences exhibit signature *i-*motif CD peaks, a positive peak at 290 nm and a negative peak at 265 nm (Fig 7A). This was confirmed via TDS where a 290 peak was observed (Fig 7B), making this wavelength region suitable for monitoring the conformational transition. To further investigate *i-*motif formation, melting experiments were performed at 290 nm. Four-stranded *i-*motif structures exhibit thermal melting at 290 nm. As seen from Fig 7C, all three C-rich DNAs exhibited cooperative melting at the signature 290 nm peak with melting temperatures of 52.01± 0.69 °C, 46.57 ± 0.73 °C and 52.11 ± 0.14 °C for XDP-dC2, XDP-dC3 and XDP-dC1 respectively. Notably, XDP-dC1 and XDP-dC2 have almost identical apparent melting temperatures whereas XDP-dC3 has the lowest melting temperature similar to our observations for the complementary G-rich strand XDP-dG3. Therefore, the CCCT interruption appears to preserve *i-*motif stability relative to the CCT interruption. In order to establish molecularity and whether the structures are intramolecular or intermolecular, further experimentation is needed.

**Fig 7:**
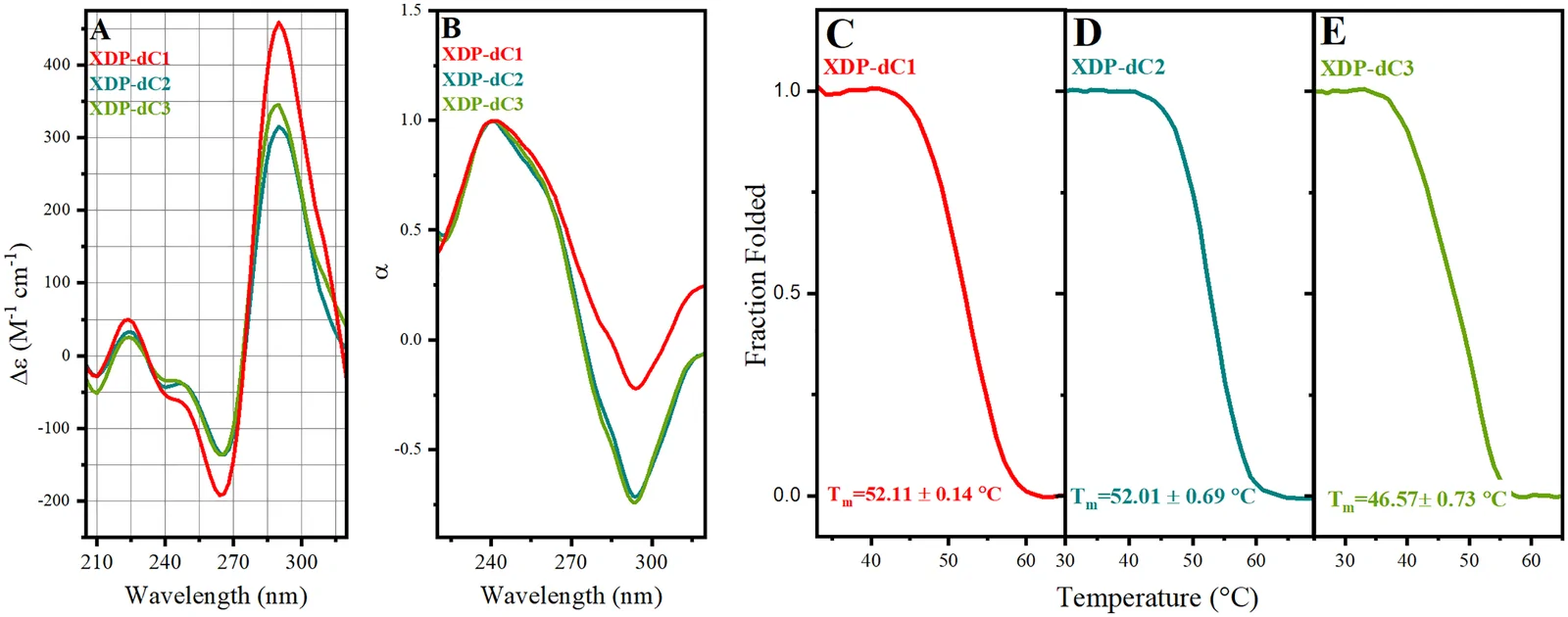
Structural characterization of the C-rich XDP oligonucleotides in 10 mM sodium phosphate buffer, 0.1mM EDTA, and 80 mM NaCl at pH 5.2: (A) CD spectra, (B) normalized thermal difference spectra, and UV thermal melting profiles of (C) XDP-dC1, (D) XDP-dC2, and (E) XDP-dC3. Thermal melting was monitored at 290 nm. The red, blue, and green curves represent XDP-dC1, XDP-dC2, and XDP-dC3, respectively. XDP-dC1 = [5’-(CCCTCT)4-3’], XDP-dC2 = [5’-(CCCTCT) *CCCT*(CCCTCT)2-3’]; and XDP-dC3 = [5’-(CCCTCT)*CCT*(CCCTCT)2-3’].

### Sequence-dependent triplex-compatible structure formation by mixtures of G-rich and C-rich DNAs of XDP repeats

Being homopurine and homopyrimidine on complementary strands, the formation of DNA triplex-compatible structures was investigated for all three sequences. DNA triplexes form when a third strand of DNA binds to the duplex DNA forming a three-stranded DNA structure. Under acidic pH, protonation of cytosine residues in the third can stabilize pyrimidine-motif triplex structures. Therefore, the CD spectra of 2:1 mixtures of XDP-dC1:XDP-dG1, XDP-dC2:XDP-dG2 and XDP-dC3:XDP-dG3 were measured in acidic versus neutral pH. The 2:1 C-rich:G-rich ratio was selected *a priori* based on the pyr•pur:pyr model. While these sequences do not strictly satisfy mirror symmetry for stable pyrimidine triplex formation, they were designed to reflect the XDP-associated SVA repeat. Thus, our goal was to asses triplex-forming propensity under biologically relevant conditions. As seen from Fig 8, for XDP-dC1:XDP-dG1 and XDP-dC2:XDP-dG2, the spectra are not superimposed at pH 5.6 and pH 7 with different peaks. At pH 5.6, a 285 nm positive peak, a 215 nm negative peak and a zero crossing are seen, reflective of pyrimidine triplex DNA formation, whereas at pH 7, the spectra exhibit positive ~270 nm and negative ~240 nm peaks [42,43] The 2:1 mixture of XDP-dC3:XDP-dG3, however, does not differ at pH 5.6 and pH 7, positive ~270 nm and negative ~240 nm peaks are seen at both pHs, showing duplex B-form DNA formation regardless of the higher concentration of the C-rich strand and acidic pH. Furthermore, the weighted arithmetic sum of the spectra of the individual strands for all three sequences studied were not superimposed with the observed spectra in Fig 8, pointing towards secondary structure formation at both pHs (S2 Fig.). Although, the signature CD spectra characteristics for pyrimidine triplexes including the 216 nm peak, the zero point crossing and the absence of typical B-DNA signals are only seen for 2:1 XDP-dC1:XDP-dG1 and 2:1 XDP-dC2:XDP-dG2, further studies are necessary for elucidating the exact structure and the presence of other folding patterns such as *i-*motifs and duplexes in solution.

**Fig 8:**
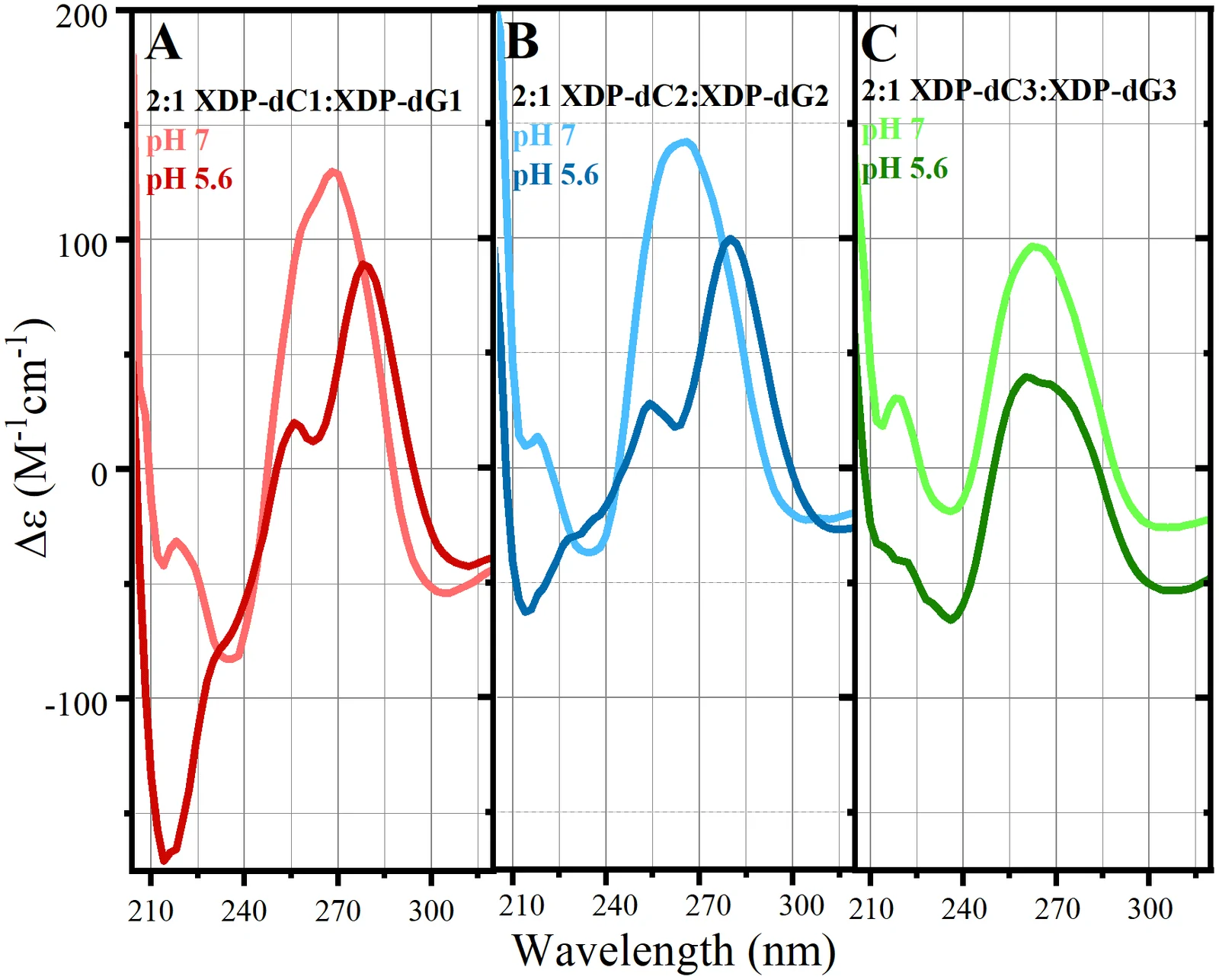
CD spectra of 2:1 C-rich:G-rich strand mixtures: (A) XDP-dC1:XDP-dG1, (B) XDP-dC2:XDP-dG2, and (C) XDP-dC3:XDP-dG3. Measurements were performed in 10 mM sodium phosphate buffer containing 0.1 mM EDTA and 150 mM NaCl at pH 5.6 and pH 7.0. Each mixture had a total strand concentration of 5 μM, comprising 3.33 μM C-rich strand and 1.67 μM G-rich strand. The dark-colored and light-colored curves represent measurements at pH 5.6 and pH 7.0, respectively. XDP-dG1 = [5’-(AGAGGG)4-3’], XDP-dG2 = [5’-(AGAGGG)2*AGGG*(AGAGGG)-3’]; and XDP-dG3 = [5’-(AGAGGG)2*AGG*(AGAGGG)-3’] and XDP-dC1 = [5’-(CCCTCT)4-3’], XDP-dC2 = [5’-(CCCTCT)*CCCT*(CCCTCT)2-3’]; and XDP-dC3 = [5’-(CCCTCT)*CCT*(CCCTCT)2-3’].

## Discussion

Repeat expansions of difference sequence motifs in a given gene can lead to either dramatic disease or be innocuous [30,44–46]. Specifically, depending upon the sequence of the expanded motif, there can be disease or no disease. This has been shown to arise at least at the *RFC1* and *FGF14* loci, where numerous forms of repeat expansions can occur at precisely the same genic location and only one motif leads to disease. The current thinking, supported by some data, is that the ability of the repeating sequence to form unusual structures (triplex and/or quadruplex) appears to be the determining factor [30,47].

Similar to the mDRILS of XDP/*TAF1*-SVA repeat, examined here, mDRILS have been identified in the expanded repeat of the *FGF14* gene and are suspected of altering disease attributes of SCA27B [48]. *In- vivo* evidence for the formation of unusual DNA structures at disease loci is sparse. Slipped-DNAs formed at the mutant *DMPK* gene have been identified in post-mortem patient tissues [49]. Slipped-DNAs are present as clusters of slip-outs along a DNA, with each slip-out having 1–100 extrahelical repeats.

The allelic levels of slipped-DNA-containing molecules were significantly greater in the heart over the cerebellum (relative to genomic equivalents of pre-IP input DNA) of a DM1 individual, an enrichment consistent with increased allelic levels of slipped-DNA structures in tissues having greater levels of CTG instability. Surprisingly, this supports the formation of slipped-DNAs as persistent mutation products of repeat instability, and not merely as transient mutagenic intermediates. These findings further our understanding of the processes of mutation and genetic variation. Somatic expansions of the XDP/*TAF1*-SVA [6] and SCA27/*FGF14* repeats [50,51] are tissue-specific [6,50,51], similar to HD, DM1, and various spinocerebellar ataxias [52–55], where this tissue-specific instability in HD [56–58] and DM1 [59] are now resolved at the cell-type level, are likely linked to driving early disease onset and more severe progression [50].

Here we structurally characterized the non-B DNA structures assumed by the canonical XDP-linked *TAF1*-SVA repeat d(AGAGGG)n and its mosaic derivatives with the two most prominent mDRILS. We have shown that the canonical repeat d(AGAGGG)4 (XDP-dG1) and one of the mDRILS with an ***AGGG*** insertion (XDP-dG2) can form G-quadruplexes in the presence of K^+^, in contrast, the derivative with the ***AGG*** mDRILS insertion (XDP-dG3) has minimal or transient G-quadruplex formation [14]. Notably, Laß et al. have previously shown that mosaic frequencies of the AGGG, but not the AGG, appeared to stabilize the XDP repeat from repeat instability and correlated with delayed AAO [14,15]. Being homopurinic, the formation of (GA)n duplexes is also probable in the G-rich sequences. The formation of cooperatively melting structures by (GA)n strands, even without the complementary pyrimidine strands, (TC)n, has been previously shown [28,60]. Using CD spectroscopy, UV melting transitions, TDS spectra and TMPyP4 G-quadruplex ligand binding studies, we have shown that in the presence of K^+^, XDP-dG1 and XDP-dG2 form a mixture of G-quadruplexes and GA duplexes exhibiting a high level of polymorphism. Rather than a G-quadruplex, GA duplexes are likely the prominent conformation assumed by XDP-dG3 in K^+^. The minimal effect of cation type on the CD spectra of XDP-dG3, as seen in Fig. 2, has been previously reported for other GA duplexes [28–30]. The higher tendency of the ***AGGG*** mDRILS XDP-dG2 for G-quadruplex formation may lie in its higher guanine-to-adenine ratio in comparison with the two other G-rich sequences, having only the canonical (AGAGGG)4 repeat, XDP-dG1, and the ***AGG*** mDRILS XDP-dG3. While XDP-dG1 and XDP-dG3 have identical guanine to adenine ratios, d(AGAGGG)4 (XDP-dG1) is more prone to G-quadruplex formation due to a higher number of guanine bases in comparison with XDP-dG3. It is also noteworthy that XDP-dG1 and XDP-dG2 have 4 full GGG tracts while XDP-dG3 has 3 GGG tracts and 1 GG tract. Furthermore, combined effects of G-tract organization, interruption position, intervening nucleotide composition and the relative stability of competing conformations can play a role in propensity towards G-quadruplex formation. Interestingly, unlike XDP-dG3, the RNA version of the mDRILS with an ***AGG*** insertion, XDP-rG3, was shown to form stable G-quadruplex structures with an unusual folding pattern, which shows that the presence of 2’-OH groups can stabilize G-quadruplex formation in sequences with a lower G:A ratio [61]. We know that the length and sequence of the intervening bases that link G-tracts in G-rich sequences seem to exert considerable effects on the formation of different topologies [19,20,62]. Given the structural polymorphism of G-quadruplexes and the limitations of CD and UV melting techniques, definitive resolution of molecularity, polarity and heterogeneity remains to be elucidated in dedicated studies combining electrophoretic, thermodynamic and higher-resolution structural approaches. We note that the XDP repeats, like *C9orf72* repeats can form heterogenous mixtures of unusual DNA conformations [63–65].

The formation of another form of non-B DNA four-stranded structure called the *i-*motif is subsequently investigated in the complementary d(CCCTCT)4 strands (XDP-dC1) in addition to the sequence containing an insertion of **CCCT** (XDP-dC2) and an insertion of **CCT**(XDP-dC3). While *i*-motif-compatible structure formation under slightly acidic pH is seen in all three sequences, XDP-dC3 exhibits the lowest thermal stability. Our observation that the highest propensity for the formation of stable G-quadruplex/*i-*motif is for mDRILS with (***AGGG***)•(***CCCT***) is in tune with the previous finding that these mDRILS could stabilize repeat lengths between parent-to-offspring transmissions [15]. That we find the XDP-linked *TAF1*-SVA repeats to have variable abilities to assume higher-order nucleic acid structures depending upon the presence and type of mDRILS, may suggest that such variation of G- and C-rich regions that are capable of forming G-quadruplex/*i-*motifs, are prevalent in regulatory elements of the genomic DNA, especially near transcriptional start sites [21]. G-quadruplex/*i-*motif formation at the same genomic DNA locations can be mutually exclusive, setting the stage for the efficient coordination of counter biological activities of G-quadruplexes and *i-*motifs [66]. Therefore, it can be hypothesized that the formation of non-B structures in the RNA can be linked to the observation of lower levels of *TAF1* transcript due to alternative splicing and non-sense mediated decay of intron-retained mRNA [13,67]. The former may be due to RNA foci formation, which in turn is dependent upon the type of RNA secondary structures formed. As shown here, mosaic insertions of mDRILS have direct effects on the folding patterns of both the DNA and RNA and may therefore play a role in pathogenesis.

Given that the XDP-linked repeats are homopurine/homopyrimidine, the formation of DNA triplexes was deemed possible. Interestingly, under midly slightly acidic pH, mixtures of 2:1 XDP-dC2:XDP-dG2 [with an (***AGGG***)•(***CCCT***) mDRILS] and XDP-dC1:XDP-dG1 (canonical) were shown to form compatible structures with pyrimidine-motif DNA triplexes, the sequences [with an (***AGG***)•(***CCT***) mDRILS], 2:1 mixture of XDP-dC3: XDP-dG3, did not support triplex formation, showing the variable ability for triple-stranded DNA formation based on sequence. Base composition and sequence order, alongside environmental factors, play key roles in triplex formation and stability [25,68–70]. The absence of a clear triplex-compatible structure by the (***AGG***)•(***CCT***) mDRILS mixture of XDP-linked repeats reflect the combined effects of sequence order, interruption of mirror symmetry and the ability to form continuous favourable base triads. Regarding molecularity and polarity of triplexes, two pyrimidine strands in a pyr:pur:pyr triplex are antiparallel to each other. Excesses of the complementary pyrimidine strand will form stable triplexes if its sequence corresponds to a mirror repeat. The mirror repeat aspect is met by the non-interrupted XDP repeat, but not by either mDRILS, which could reduce the stability, pH-sensitivity, or completeness of triplex formation. Our goal was to assess triplex-forming propensity of genetically- and clinically-relevant sequence variations, under biologically relevant conditions. In the case of XDP-dC1:XDP-dG1 and XDPdC2:XDP-dG2, several observations support triplex-compatible structure formation, possibly via local symmetry or partial binding. These include pH sensitivity, characteristic pyr•pur:pyr CD peaks and spectra that do not correspond to the weighted sum of C-rich and G-rich controls (S2 Fig.). The weaker triplex signatures of XDP-dC2:XDP-dG2 may result from repeat interruptions that disrupt mirror symmetry. Longer tract expansions, not readily assessed by CD or melting, with variable levels of interrupting mDRILS may alter triplex formation. Considering that the repeat itself regulates transcription, possible DNA triplex formation by disease-relevant length repeats can be linked to errors in the transcription process [3,71]. Notably, stressed cell/neuron environments may lead to pH imbalances — resulting in mildly acidic intranuclear pH, which may be sufficient to modulate the equilibrium between alternative repeat structures such as *i-*motifs and triplexes [24,72]. Possible formation of these non-B DNA structures may contribute to unusual interactions with both somatic repeat instability, TAF1 transcription, TAF1 splicing, and RAN-translation. Moreover, these possible mechanisms would be intensified by the instability of somatic repeats, which affects not only the AAO but also the pathogenic characteristics’ tissue specificity [4]. Distinct pathological mechanisms are not mutually exclusive and neurodegeneration can be driven as a synergistic effect of various toxic mechanisms [73].

It was recently shown that quadruplex formation by the pathogenic XDP repeats in intron 32 of *TAF1* blocks full-length *TAF1* transcription across the repeats, yielding increased transcripts truncated at or near the SVA [17]. Furthermore, the ZNF91 protein was shown to bind to DNA sequences capable of forming quadruplex structures, including the pathogenic XDP repeat [16]. ZNF91 binding appeared to block the DNA from forming quadruplex structures, minimizing the production of the XDP-pathogenic truncated *TAF*1 transcripts [16]. These recent studies did not consider either the formation of triplex DNAs or *i-*motifs, nor did they consider mDRILS. Our findings suggest that AGGG mDRILS promote G4/triplex formation, while AGG mDRILS reduce them, revealing how subtle repeat variations may cause significant structural consequences. While these findings, limited to spectroscopic data on short non-disease length repeats, offer insights at a global level into how mDRILS may modulate disease pathogenesis, additional biological evidence of repeats with disease relevant length needed to strengthen this structural connection to the physiological properties. Our findings suggest that the role of mDRILS on the ability to form quadruplexes, triplexes, and *i-*motifs, may alter both perturbed transcription of the pathogenic XDP *TAF1*, and may, at a somatically mosaic level, also alter repeat instability, such that mDRILS can delay age at onset.

## Experimental procedures

### Sample preparation

The oligonucleotides listed in Table 1 were purchased from IDT (Iowa, United States), and ACGT corp. (Toronto, ON, Canada). The concentrations were determined by recording their absorbance at 260nm using the molar extinction coefficients listed in Table 1. The guanine-rich oligos were dissolved in 100 mM KCl, 10 mM Tris-HCl, and 0.1 mM EDTA (pH 7.4) or 100 mM LiCl, 10 mM Tris-HCl, and 0.1 mM EDTA (pH 7.4), and the cytosine-rich oligos were dissolved in 10 mM sodium phosphate, 0.1 mM EDTA, and 80 mM NaCl (pH 5.2 or 5.6) unless otherwise stated. The samples were heated to 95 °C and allowed to cool down to room temperature overnight.

### Circular dichroism spectroscopy (CD)

Circular dichroism (CD) spectra were measured using a Jasco J-810 spectropolarimeter, using 5µM sequence samples dissolved in the stated buffer. For the CD spectra, three different samples were prepared and contained in a 0.1 cm pathlength cuvette and the spectra were measured over the wavelength range of 190–330 nm at a scan rate of 200 nm/min. The spectra shown here are the average of three consecutive scans, acquired from a single sample preparation and were corrected against the corresponding buffer. CD data were converted to molar circular-dichroic absorption (Δε) based on strand concentration (C) using the equation:


$$\begin{array}{c} \Delta \varepsilon =\theta /(\text{3}\text{2}\text{9}\text{8}\text{0}*C*l) \end{array}$$ (1)

where *θ* is the measured ellipticity value [mdeg], *C* is the molar strand concentration[M], and *l* is the optical path of a used cuvette [cm].

### Thermal difference spectra

All experiments were performed on a Varian Cary 100 UV–Vis spectrophotometer (Mulgrave, Australia) using cuvettes with an optical path length of 1 cm in a Peltier multi-cell (6*6) holder. Absorbance spectra were recorded in the 220–340 nm range, with a scan speed of 200 nm/min, with a data interval of 1 nm. In all cases, the sequence concentration was set at 0.005 mM, and they were dissolved in 10 mM Tris–HCl, 0.1 mM EDTA (pH 7.4) and 100 mM KCl or LiCl, or 10 mM sodium phosphate, 0.1 mM EDTA, and 80 mM NaCl (pH 5.2).These thermal difference spectra were then normalized in order to simplify the comparison by dividing the raw data by its maximum positive value, where that the highest positive peak gets a Y-value of +1 [32]. The UV absorption measurements used to generate the TDS profiles were repeated at least twice using separate sample preparations.

### UV thermal melting

UV thermal melting experiments were monitored on a Varian Cary 100 (Mulgrave, Australia) spectrometer by recording changes in the UV absorption at either 260 nm or 295 nm for G-rich sequences and 290 nm for C-rich sequences using a 1 cm path length cuvette. The heating rate was adjusted to$\frac{\text{1}\circ \text{C}}{min}$ with data intervals of 0.5∘C. The T_m_ values were determined from dual baseline-corrected 1−0 normalized curves (1- native and 0- denatured forms). For melting curves with two resolved transitions, each transition was separately baseline-corrected and normalized. The apparent T_m_ for each transition was defined as the temperature at which the normalized folded fraction was 0.5 [27]. Thermal-melting experiments were repeated at least twice using separate sample preparations.

### Soret band shift

A guanine-rich deoxyribonucleicacid or ribonucleicacid (XDP-G1, XDP-G2, XDP-G3 and r(XDP-G2)) with the concentration of 100 μM, folded in 10 mM Tris-HCl, 0.1 mM EDTA, and 100 mM KCl at pH 7.4. The 7 μM TMPyP4 solution in the cuvette was prepared in the same buffer. Therefore, the buffer composition and ionic strength were maintained consistently across all TMPyP4 titration experiments. The absorption spectra were collected with a Varian Cary 100 UV–Vis spectrophotometer from 350 nm to 520 nm using cuvettes with an optical path length of 1 cm. The concentration of free TMPyP4 was determined using an extinction coefficient of 2.26 × 10^5^ M^−1^ cm^−1^ and absorbance values at 424 nm. The titration was stopped when three successive additions of the sample resulted in no further shift of the Soret band. The TMPyP4-binding titration was performed once for each oligonucleotide sample.

All values were corrected for the dilution effect. The fraction (α) of TmPyP4 bound was determined as follows:


$$\begin{array}{c} \alpha =({AbsTMPyP\text{4}}_{free}-{Abs}_{mixture})/({AbsTMPyP\text{4}}_{free}-{Abs}_{bound} \end{array}$$ (2)

where Abs TMPyP4_free_ is the absorbance of the free TMPyP4 in the absence of any added DNA, Abs_mixture_ is the absorbance at any point after the beginning of the addition of the DNA and Abs_bond_ is the absorbance of fully bound TmPyP4 measured at 424 nm (the Soret maxima for TMPyP4). Concentrations of free TMPyP4 ([TMPyP4]_free_) and the concentration of bound TMPyP4 ([TMPyP4]_bond_) were calculated as follows:


$$\begin{array}{c} {\left[TMPyP\text{4}\right]}_{free}=[TMPyP\text{4}{]}_{corrected}(\text{1}-\alpha ) \end{array}$$ (3)

$$\begin{array}{c} {\left[TMPyP\text{4}\right]}_{bound}={\left[TMPyP\text{4}\right]}_{corrected}-[TMPyP\text{4}{]}_{free} \end{array}$$ (4)

where [TMPyP4]_corrected_ represents the concentration of TMPyP4 corrected for the volume changes due to the titrated DNA [74].

The percentage of hypochromicity of the Soret band is calculated as follows [75]:


$$\begin{array}{c} \%Hypochromicity=\left[\left(\varepsilon_{free}-\varepsilon_{bound}\right)\right]*\text{1}\text{0} \end{array}$$ (5)

Where


$$\begin{array}{c} \varepsilon_{bound}={AbsTMPyP\text{4}}_{bound}/{\left[TMPyP\text{4}\right]}_{free} \end{array}$$ (6)

### Triplex formation

The single-stranded C-rich and the corresponding complementary G-rich oligos were mixed together (separately for each sequence) in weighed proportions to give XDP-dC: XDP-dG mixtures with molar ratios of 2:1. They were dissolved in 10 mM sodium phosphate buffer and 150 mM NaCl (pH 5.6 and pH 7) with a total DNA strand concentration of 5 μM, comprising 3.33 μM C-rich strand and 1.67 μM G-rich strand. To overcome intra-strand pairing the samples were heated to 95°C and allowed to cool overnight.

### Calculation of the binding constant

The Soret band shift upon TMPyP4 binding with the oligonucleotides was used to calculate the binding constants K_app_ using the Hill equation [76].


$$\begin{array}{c} \alpha =\frac{\left[DNA\right]}{k_{d}^{n}+[{DNA]}^{n}} \end{array}$$ (7)

Where [DNA] is the total DNA concentration, K_d_ is the dissociation constant and n is the Hill coefficient. K_app_ is the inverse of K_d_.

## Supporting information

S1 Fig Derivative of Absorbance vs. Temperature derived from the thermal melting curves for XDP-G1, XDP-G2 and XDP-G3 in KCl. (TIFF)

S2 Fig CD spectra of weighted sum of spectra of individual strands (black) and pH-induced complex spectra (red) for (A) 0.66 XDPdC1:0.33XDPdG1 (B) 0.66 XDPdC2:0.33XDPdG2 (C) 0.66 XDPdC3:0.33XDPdG3 in 10 mM Sodium Phosphate buffer, 0.1 mM EDTA, 150 mM NaCl at pH 5.6. (TIFF)

S3 Fig Contour Plots for (A) two singular values (B) three singular values (C) four singular values (D) five singular values. (TIFF)

S4 Fig First six weighted basis spectra plots. (TIFF)

S1 Table Singular values and autocorrelation values from SVD analysis of titration data from Fig. 5B. (DOCX)

S1 File Plos one raw data. (ZIP)
