# Supplementary material for: Mosaic divergent repeat interruptions of motif length and sequence, mDRILS, of XDP-linked repeats affect formation of DNA/RNA triplexes & quadruplexes
Source: PLoS One. 2026 Oct 27;21(10):e0360550. doi: 10.1371/journal.pone.0360550 (PMC13712557; doi:10.1371/journal.pone.0360550)
Supplement: Supplementary file 1 [file pone.0360550.s005.docx]

| **Number of values** | **Singular values** | **Autocorrelation values** | |
| --- | --- | --- | --- |
|  |  | **U** | **V** |
| **1** | 18.4110 | 0.9995 | 0.9905 |
| **2** | 4.0893 | 0.9961 | 0.8461 |
| **3** | 0.3168 | 0.9912 | -0.1655 |
| **4** | 0.2010 | 0.9924 | 0.5972 |
| **5** | 0.0417 | 0.9607 | -0.4437 |
| **6** | 0.0156 | 0.8444 | 0.4483 |
| **7** | 0.0075 | 0.5713 | 0.0622 |
| **8** | 0.0068 | 0.3466 | -0.4250 |
| **9** | 0.0052 | 0.2260 | -0.6987 |
| **10** | 0.0047 | 0.0665 | -0.4797 |
| **11** | 0.0044 | 0.0135 | -0.4383 |
