# Supplementary material for: Mosaic divergent repeat interruptions of motif length and sequence, mDRILS, of XDP-linked repeats affect formation of DNA/RNA triplexes & quadruplexes
Source: PLoS One. 2026 Oct 27;21(10):e0360550. doi: 10.1371/journal.pone.0360550 (PMC13712557; doi:10.1371/journal.pone.0360550)
Supplement: Supplementary file 2 [file pone.0360550.s006.zip › PLOS one raw data/Drug Binding/drug binding.docx]

1-G

|  | Abs |  |  |  |  |  |  |  |  |
| --- | --- | --- | --- | --- | --- | --- | --- | --- | --- |
| 300 | 0.294 | 0.2 | 0.192 | 0.211 | 0.214 | 0.237 | 0.232 | 0.266 | 0.275 |
| 301 | 0.296 | 0.198 | 0.189 | 0.206 | 0.208 | 0.23 | 0.223 | 0.254 | 0.262 |
| 302 | 0.299 | 0.196 | 0.186 | 0.201 | 0.202 | 0.222 | 0.212 | 0.241 | 0.247 |
| 303 | 0.3 | 0.195 | 0.183 | 0.197 | 0.197 | 0.216 | 0.206 | 0.232 | 0.237 |
| 304 | 0.302 | 0.194 | 0.182 | 0.194 | 0.193 | 0.211 | 0.199 | 0.223 | 0.227 |
| 305 | 0.305 | 0.193 | 0.18 | 0.191 | 0.19 | 0.206 | 0.193 | 0.215 | 0.219 |
| 306 | 0.307 | 0.193 | 0.179 | 0.189 | 0.187 | 0.203 | 0.189 | 0.21 | 0.213 |
| 307 | 0.309 | 0.192 | 0.178 | 0.187 | 0.185 | 0.2 | 0.186 | 0.206 | 0.208 |
| 308 | 0.312 | 0.193 | 0.178 | 0.187 | 0.183 | 0.198 | 0.184 | 0.203 | 0.204 |
| 309 | 0.313 | 0.192 | 0.177 | 0.186 | 0.183 | 0.197 | 0.182 | 0.2 | 0.201 |
| 310 | 0.315 | 0.193 | 0.178 | 0.186 | 0.182 | 0.196 | 0.18 | 0.198 | 0.199 |
| 311 | 0.317 | 0.194 | 0.178 | 0.185 | 0.181 | 0.195 | 0.179 | 0.197 | 0.198 |
| 312 | 0.318 | 0.194 | 0.178 | 0.185 | 0.181 | 0.195 | 0.179 | 0.196 | 0.197 |
| 313 | 0.32 | 0.194 | 0.178 | 0.185 | 0.181 | 0.194 | 0.178 | 0.196 | 0.196 |
| 314 | 0.321 | 0.195 | 0.179 | 0.185 | 0.181 | 0.194 | 0.178 | 0.195 | 0.195 |
| 315 | 0.323 | 0.195 | 0.179 | 0.185 | 0.181 | 0.194 | 0.178 | 0.194 | 0.195 |
| 316 | 0.324 | 0.196 | 0.179 | 0.185 | 0.181 | 0.194 | 0.178 | 0.194 | 0.194 |
| 317 | 0.326 | 0.196 | 0.179 | 0.186 | 0.181 | 0.194 | 0.178 | 0.194 | 0.194 |
| 318 | 0.326 | 0.196 | 0.179 | 0.186 | 0.181 | 0.194 | 0.177 | 0.194 | 0.194 |
| 319 | 0.328 | 0.197 | 0.18 | 0.186 | 0.181 | 0.195 | 0.178 | 0.194 | 0.194 |
| 320 | 0.329 | 0.198 | 0.18 | 0.185 | 0.181 | 0.194 | 0.178 | 0.193 | 0.193 |
| 321 | 0.33 | 0.197 | 0.18 | 0.186 | 0.181 | 0.194 | 0.178 | 0.194 | 0.194 |
| 322 | 0.332 | 0.198 | 0.18 | 0.187 | 0.182 | 0.195 | 0.178 | 0.194 | 0.194 |
| 323 | 0.332 | 0.199 | 0.181 | 0.187 | 0.182 | 0.195 | 0.178 | 0.194 | 0.194 |
| 324 | 0.333 | 0.199 | 0.182 | 0.187 | 0.182 | 0.195 | 0.178 | 0.195 | 0.194 |
| 325 | 0.335 | 0.199 | 0.181 | 0.188 | 0.182 | 0.195 | 0.178 | 0.195 | 0.194 |
| 326 | 0.336 | 0.201 | 0.183 | 0.189 | 0.183 | 0.196 | 0.179 | 0.196 | 0.195 |
| 327 | 0.337 | 0.201 | 0.183 | 0.188 | 0.183 | 0.196 | 0.179 | 0.196 | 0.195 |
| 328 | 0.34 | 0.201 | 0.183 | 0.189 | 0.183 | 0.197 | 0.18 | 0.196 | 0.195 |
| 329 | 0.341 | 0.202 | 0.184 | 0.19 | 0.184 | 0.197 | 0.18 | 0.196 | 0.196 |
| 330 | 0.342 | 0.203 | 0.184 | 0.19 | 0.185 | 0.197 | 0.181 | 0.196 | 0.196 |
| 331 | 0.344 | 0.203 | 0.185 | 0.19 | 0.185 | 0.198 | 0.181 | 0.197 | 0.197 |
| 332 | 0.308 | 0.191 | 0.174 | 0.177 | 0.175 | 0.184 | 0.173 | 0.186 | 0.185 |
| 333 | 0.31 | 0.192 | 0.175 | 0.177 | 0.175 | 0.184 | 0.173 | 0.187 | 0.185 |
| 334 | 0.311 | 0.193 | 0.175 | 0.178 | 0.176 | 0.184 | 0.174 | 0.187 | 0.186 |
| 335 | 0.313 | 0.194 | 0.176 | 0.178 | 0.177 | 0.185 | 0.174 | 0.188 | 0.186 |
| 336 | 0.315 | 0.194 | 0.177 | 0.179 | 0.178 | 0.186 | 0.175 | 0.189 | 0.187 |
| 337 | 0.317 | 0.196 | 0.178 | 0.18 | 0.178 | 0.187 | 0.176 | 0.19 | 0.189 |
| 338 | 0.319 | 0.197 | 0.179 | 0.181 | 0.179 | 0.187 | 0.177 | 0.191 | 0.189 |
| 339 | 0.322 | 0.198 | 0.18 | 0.182 | 0.18 | 0.188 | 0.178 | 0.192 | 0.19 |
| 340 | 0.325 | 0.199 | 0.181 | 0.183 | 0.181 | 0.189 | 0.179 | 0.193 | 0.191 |
| 341 | 0.327 | 0.201 | 0.182 | 0.184 | 0.182 | 0.19 | 0.18 | 0.193 | 0.192 |
| 342 | 0.33 | 0.202 | 0.183 | 0.185 | 0.183 | 0.191 | 0.181 | 0.194 | 0.192 |
| 343 | 0.332 | 0.203 | 0.184 | 0.186 | 0.183 | 0.192 | 0.181 | 0.195 | 0.194 |
| 344 | 0.336 | 0.204 | 0.185 | 0.187 | 0.185 | 0.193 | 0.183 | 0.196 | 0.195 |
| 345 | 0.338 | 0.206 | 0.187 | 0.188 | 0.186 | 0.194 | 0.184 | 0.198 | 0.196 |
| 346 | 0.341 | 0.207 | 0.188 | 0.19 | 0.187 | 0.195 | 0.185 | 0.199 | 0.197 |
| 347 | 0.344 | 0.209 | 0.189 | 0.191 | 0.188 | 0.196 | 0.186 | 0.2 | 0.198 |
| 348 | 0.348 | 0.21 | 0.19 | 0.192 | 0.189 | 0.197 | 0.187 | 0.201 | 0.199 |
| 349 | 0.35 | 0.211 | 0.191 | 0.193 | 0.19 | 0.199 | 0.188 | 0.202 | 0.201 |
| 350 | 0.352 | 0.212 | 0.192 | 0.194 | 0.191 | 0.2 | 0.189 | 0.203 | 0.202 |
| 351 | 0.354 | 0.214 | 0.193 | 0.195 | 0.192 | 0.2 | 0.19 | 0.204 | 0.203 |
| 352 | 0.358 | 0.215 | 0.195 | 0.196 | 0.193 | 0.202 | 0.191 | 0.205 | 0.204 |
| 353 | 0.36 | 0.216 | 0.196 | 0.197 | 0.194 | 0.203 | 0.193 | 0.206 | 0.205 |
| 354 | 0.362 | 0.217 | 0.197 | 0.198 | 0.195 | 0.204 | 0.194 | 0.207 | 0.206 |
| 355 | 0.364 | 0.219 | 0.198 | 0.199 | 0.196 | 0.205 | 0.195 | 0.209 | 0.208 |
| 356 | 0.366 | 0.22 | 0.199 | 0.2 | 0.197 | 0.206 | 0.196 | 0.209 | 0.208 |
| 357 | 0.369 | 0.221 | 0.2 | 0.201 | 0.198 | 0.207 | 0.197 | 0.211 | 0.209 |
| 358 | 0.37 | 0.222 | 0.201 | 0.202 | 0.199 | 0.207 | 0.198 | 0.211 | 0.211 |
| 359 | 0.372 | 0.223 | 0.201 | 0.203 | 0.2 | 0.208 | 0.198 | 0.212 | 0.211 |
| 360 | 0.374 | 0.224 | 0.202 | 0.204 | 0.2 | 0.209 | 0.199 | 0.213 | 0.212 |
| 361 | 0.376 | 0.225 | 0.203 | 0.205 | 0.201 | 0.21 | 0.2 | 0.214 | 0.213 |
| 362 | 0.378 | 0.226 | 0.204 | 0.205 | 0.202 | 0.21 | 0.2 | 0.215 | 0.214 |
| 363 | 0.38 | 0.227 | 0.204 | 0.206 | 0.202 | 0.211 | 0.201 | 0.215 | 0.214 |
| 364 | 0.382 | 0.227 | 0.205 | 0.206 | 0.202 | 0.211 | 0.201 | 0.216 | 0.215 |
| 365 | 0.384 | 0.228 | 0.206 | 0.207 | 0.203 | 0.212 | 0.202 | 0.216 | 0.215 |
| 366 | 0.387 | 0.229 | 0.207 | 0.208 | 0.204 | 0.212 | 0.202 | 0.216 | 0.215 |
| 367 | 0.389 | 0.23 | 0.207 | 0.208 | 0.204 | 0.212 | 0.202 | 0.217 | 0.216 |
| 368 | 0.391 | 0.231 | 0.208 | 0.209 | 0.205 | 0.213 | 0.203 | 0.217 | 0.216 |
| 369 | 0.393 | 0.232 | 0.208 | 0.209 | 0.205 | 0.213 | 0.203 | 0.217 | 0.216 |
| 370 | 0.396 | 0.233 | 0.209 | 0.21 | 0.205 | 0.214 | 0.204 | 0.218 | 0.217 |
| 371 | 0.398 | 0.234 | 0.21 | 0.211 | 0.206 | 0.214 | 0.204 | 0.218 | 0.217 |
| 372 | 0.4 | 0.235 | 0.211 | 0.211 | 0.206 | 0.215 | 0.204 | 0.219 | 0.217 |
| 373 | 0.402 | 0.236 | 0.211 | 0.212 | 0.207 | 0.215 | 0.205 | 0.219 | 0.217 |
| 374 | 0.405 | 0.236 | 0.212 | 0.212 | 0.207 | 0.215 | 0.205 | 0.219 | 0.218 |
| 375 | 0.406 | 0.235 | 0.21 | 0.21 | 0.205 | 0.213 | 0.203 | 0.217 | 0.216 |
| 376 | 0.407 | 0.235 | 0.21 | 0.211 | 0.205 | 0.213 | 0.204 | 0.217 | 0.216 |
| 377 | 0.409 | 0.236 | 0.211 | 0.211 | 0.206 | 0.214 | 0.204 | 0.217 | 0.216 |
| 378 | 0.412 | 0.238 | 0.212 | 0.212 | 0.206 | 0.215 | 0.205 | 0.218 | 0.217 |
| 379 | 0.414 | 0.239 | 0.213 | 0.213 | 0.207 | 0.215 | 0.205 | 0.219 | 0.217 |
| 380 | 0.417 | 0.239 | 0.214 | 0.213 | 0.207 | 0.216 | 0.205 | 0.218 | 0.218 |
| 381 | 0.42 | 0.241 | 0.215 | 0.214 | 0.209 | 0.216 | 0.206 | 0.219 | 0.218 |
| 382 | 0.423 | 0.242 | 0.216 | 0.215 | 0.209 | 0.217 | 0.207 | 0.22 | 0.219 |
| 383 | 0.427 | 0.243 | 0.217 | 0.216 | 0.209 | 0.217 | 0.207 | 0.22 | 0.219 |
| 384 | 0.431 | 0.245 | 0.218 | 0.217 | 0.21 | 0.218 | 0.207 | 0.22 | 0.219 |
| 385 | 0.437 | 0.247 | 0.219 | 0.217 | 0.211 | 0.218 | 0.208 | 0.221 | 0.22 |
| 386 | 0.442 | 0.249 | 0.221 | 0.218 | 0.212 | 0.219 | 0.209 | 0.222 | 0.221 |
| 387 | 0.45 | 0.252 | 0.223 | 0.22 | 0.212 | 0.22 | 0.209 | 0.222 | 0.221 |
| 388 | 0.457 | 0.254 | 0.225 | 0.221 | 0.213 | 0.221 | 0.21 | 0.223 | 0.222 |
| 389 | 0.466 | 0.257 | 0.226 | 0.223 | 0.215 | 0.221 | 0.211 | 0.223 | 0.222 |
| 390 | 0.478 | 0.262 | 0.23 | 0.225 | 0.216 | 0.223 | 0.211 | 0.224 | 0.223 |
| 391 | 0.49 | 0.266 | 0.232 | 0.226 | 0.217 | 0.224 | 0.212 | 0.225 | 0.223 |
| 392 | 0.507 | 0.272 | 0.236 | 0.229 | 0.219 | 0.225 | 0.213 | 0.225 | 0.224 |
| 393 | 0.522 | 0.277 | 0.239 | 0.231 | 0.221 | 0.226 | 0.214 | 0.226 | 0.224 |
| 394 | 0.542 | 0.283 | 0.244 | 0.234 | 0.223 | 0.228 | 0.215 | 0.227 | 0.225 |
| 395 | 0.56 | 0.29 | 0.248 | 0.237 | 0.225 | 0.229 | 0.216 | 0.228 | 0.226 |
| 396 | 0.584 | 0.298 | 0.254 | 0.24 | 0.227 | 0.231 | 0.218 | 0.228 | 0.227 |
| 397 | 0.604 | 0.305 | 0.258 | 0.243 | 0.23 | 0.233 | 0.219 | 0.229 | 0.227 |
| 398 | 0.63 | 0.314 | 0.265 | 0.248 | 0.233 | 0.235 | 0.22 | 0.231 | 0.229 |
| 399 | 0.655 | 0.323 | 0.271 | 0.252 | 0.236 | 0.237 | 0.222 | 0.232 | 0.23 |
| 400 | 0.68 | 0.331 | 0.277 | 0.256 | 0.239 | 0.239 | 0.224 | 0.233 | 0.231 |
| 401 | 0.708 | 0.342 | 0.284 | 0.261 | 0.242 | 0.242 | 0.226 | 0.235 | 0.232 |
| 402 | 0.739 | 0.353 | 0.291 | 0.266 | 0.246 | 0.245 | 0.229 | 0.237 | 0.234 |
| 403 | 0.774 | 0.366 | 0.301 | 0.273 | 0.251 | 0.249 | 0.232 | 0.239 | 0.236 |
| 404 | 0.805 | 0.378 | 0.309 | 0.279 | 0.256 | 0.253 | 0.235 | 0.241 | 0.238 |
| 405 | 0.846 | 0.392 | 0.319 | 0.286 | 0.262 | 0.257 | 0.238 | 0.245 | 0.241 |
| 406 | 0.885 | 0.407 | 0.33 | 0.294 | 0.267 | 0.262 | 0.242 | 0.248 | 0.244 |
| 407 | 0.934 | 0.425 | 0.342 | 0.303 | 0.274 | 0.267 | 0.246 | 0.251 | 0.247 |
| 408 | 0.989 | 0.445 | 0.356 | 0.313 | 0.282 | 0.273 | 0.251 | 0.255 | 0.251 |
| 409 | 1.052 | 0.468 | 0.372 | 0.324 | 0.29 | 0.28 | 0.257 | 0.26 | 0.256 |
| 410 | 1.118 | 0.492 | 0.388 | 0.335 | 0.299 | 0.287 | 0.263 | 0.265 | 0.26 |
| 411 | 1.195 | 0.52 | 0.408 | 0.349 | 0.309 | 0.295 | 0.269 | 0.27 | 0.265 |
| 412 | 1.255 | 0.543 | 0.423 | 0.36 | 0.318 | 0.302 | 0.275 | 0.275 | 0.27 |
| 413 | 1.343 | 0.575 | 0.446 | 0.375 | 0.33 | 0.311 | 0.283 | 0.282 | 0.276 |
| 414 | 1.425 | 0.607 | 0.467 | 0.39 | 0.341 | 0.321 | 0.29 | 0.288 | 0.282 |
| 415 | 1.505 | 0.637 | 0.488 | 0.406 | 0.353 | 0.33 | 0.298 | 0.295 | 0.289 |
| 416 | 1.584 | 0.671 | 0.512 | 0.423 | 0.366 | 0.341 | 0.308 | 0.303 | 0.297 |
| 417 | 1.651 | 0.699 | 0.531 | 0.437 | 0.378 | 0.351 | 0.316 | 0.311 | 0.304 |
| 418 | 1.714 | 0.726 | 0.552 | 0.453 | 0.391 | 0.362 | 0.326 | 0.32 | 0.312 |
| 419 | 1.773 | 0.754 | 0.573 | 0.471 | 0.406 | 0.376 | 0.338 | 0.33 | 0.322 |
| 420 | 1.809 | 0.773 | 0.588 | 0.483 | 0.417 | 0.386 | 0.347 | 0.339 | 0.331 |
| 421 | 1.831 | 0.79 | 0.603 | 0.497 | 0.43 | 0.398 | 0.359 | 0.35 | 0.342 |
| 422 | 1.835 | 0.799 | 0.613 | 0.508 | 0.441 | 0.409 | 0.37 | 0.361 | 0.352 |
| 423 | 1.832 | 0.806 | 0.621 | 0.52 | 0.453 | 0.421 | 0.382 | 0.373 | 0.364 |
| 424 | 1.811 | 0.806 | 0.625 | 0.527 | 0.461 | 0.431 | 0.392 | 0.383 | 0.374 |
| 425 | 1.768 | 0.802 | 0.628 | 0.536 | 0.473 | 0.444 | 0.406 | 0.397 | 0.389 |
| 426 | 1.731 | 0.794 | 0.628 | 0.542 | 0.481 | 0.455 | 0.418 | 0.41 | 0.401 |
| 427 | 1.67 | 0.78 | 0.625 | 0.548 | 0.491 | 0.468 | 0.433 | 0.426 | 0.417 |
| 428 | 1.601 | 0.765 | 0.62 | 0.553 | 0.499 | 0.479 | 0.447 | 0.442 | 0.433 |
| 429 | 1.521 | 0.745 | 0.614 | 0.557 | 0.508 | 0.492 | 0.462 | 0.459 | 0.451 |
| 430 | 1.437 | 0.725 | 0.606 | 0.56 | 0.516 | 0.504 | 0.477 | 0.477 | 0.47 |
| 431 | 1.351 | 0.703 | 0.598 | 0.562 | 0.524 | 0.516 | 0.492 | 0.495 | 0.488 |
| 432 | 1.26 | 0.68 | 0.588 | 0.563 | 0.531 | 0.528 | 0.508 | 0.513 | 0.508 |
| 433 | 1.171 | 0.656 | 0.577 | 0.564 | 0.538 | 0.54 | 0.523 | 0.532 | 0.528 |
| 434 | 1.081 | 0.632 | 0.566 | 0.564 | 0.544 | 0.551 | 0.539 | 0.551 | 0.548 |
| 435 | 0.994 | 0.608 | 0.554 | 0.563 | 0.549 | 0.562 | 0.553 | 0.57 | 0.568 |
| 436 | 0.917 | 0.586 | 0.543 | 0.561 | 0.553 | 0.57 | 0.565 | 0.586 | 0.586 |
| 437 | 0.845 | 0.565 | 0.532 | 0.559 | 0.556 | 0.578 | 0.576 | 0.602 | 0.603 |
| 438 | 0.77 | 0.541 | 0.519 | 0.555 | 0.557 | 0.584 | 0.587 | 0.617 | 0.62 |
| 439 | 0.707 | 0.521 | 0.506 | 0.55 | 0.557 | 0.588 | 0.595 | 0.629 | 0.633 |
| 440 | 0.642 | 0.498 | 0.492 | 0.543 | 0.555 | 0.591 | 0.602 | 0.641 | 0.647 |
| 441 | 0.594 | 0.481 | 0.482 | 0.538 | 0.553 | 0.592 | 0.606 | 0.648 | 0.656 |
| 442 | 0.54 | 0.459 | 0.466 | 0.528 | 0.547 | 0.589 | 0.607 | 0.653 | 0.662 |
| 443 | 0.49 | 0.438 | 0.45 | 0.516 | 0.539 | 0.584 | 0.604 | 0.655 | 0.666 |
| 444 | 0.455 | 0.422 | 0.438 | 0.506 | 0.531 | 0.578 | 0.601 | 0.653 | 0.665 |
| 445 | 0.42 | 0.404 | 0.423 | 0.494 | 0.521 | 0.57 | 0.594 | 0.649 | 0.662 |
| 446 | 0.389 | 0.388 | 0.409 | 0.481 | 0.509 | 0.559 | 0.585 | 0.642 | 0.655 |
| 447 | 0.361 | 0.371 | 0.394 | 0.466 | 0.496 | 0.546 | 0.572 | 0.631 | 0.646 |
| 448 | 0.34 | 0.356 | 0.381 | 0.453 | 0.483 | 0.534 | 0.561 | 0.62 | 0.635 |
| 449 | 0.316 | 0.339 | 0.364 | 0.435 | 0.466 | 0.516 | 0.544 | 0.603 | 0.618 |
| 450 | 0.299 | 0.326 | 0.351 | 0.421 | 0.451 | 0.501 | 0.529 | 0.588 | 0.603 |
| 451 | 0.28 | 0.309 | 0.335 | 0.403 | 0.433 | 0.482 | 0.508 | 0.567 | 0.582 |
| 452 | 0.264 | 0.294 | 0.319 | 0.384 | 0.414 | 0.461 | 0.487 | 0.544 | 0.559 |
| 453 | 0.252 | 0.281 | 0.305 | 0.368 | 0.396 | 0.443 | 0.467 | 0.523 | 0.537 |
| 454 | 0.241 | 0.269 | 0.291 | 0.352 | 0.379 | 0.424 | 0.447 | 0.501 | 0.515 |
| 455 | 0.232 | 0.257 | 0.279 | 0.336 | 0.362 | 0.405 | 0.427 | 0.479 | 0.493 |
| 456 | 0.222 | 0.245 | 0.265 | 0.319 | 0.344 | 0.385 | 0.405 | 0.454 | 0.467 |
| 457 | 0.215 | 0.235 | 0.253 | 0.305 | 0.328 | 0.367 | 0.385 | 0.432 | 0.445 |
| 458 | 0.208 | 0.224 | 0.241 | 0.289 | 0.31 | 0.347 | 0.364 | 0.409 | 0.42 |
| 459 | 0.202 | 0.214 | 0.229 | 0.274 | 0.293 | 0.328 | 0.343 | 0.385 | 0.395 |
| 460 | 0.198 | 0.206 | 0.22 | 0.262 | 0.28 | 0.313 | 0.326 | 0.366 | 0.376 |
| 461 | 0.193 | 0.198 | 0.21 | 0.249 | 0.266 | 0.297 | 0.309 | 0.346 | 0.355 |
| 462 | 0.189 | 0.19 | 0.201 | 0.237 | 0.253 | 0.283 | 0.292 | 0.327 | 0.335 |
| 463 | 0.186 | 0.183 | 0.193 | 0.226 | 0.241 | 0.268 | 0.276 | 0.309 | 0.316 |
| 464 | 0.183 | 0.176 | 0.184 | 0.215 | 0.227 | 0.253 | 0.259 | 0.29 | 0.295 |
| 465 | 0.18 | 0.17 | 0.177 | 0.206 | 0.217 | 0.241 | 0.246 | 0.275 | 0.28 |
| 466 | 0.178 | 0.164 | 0.169 | 0.196 | 0.206 | 0.228 | 0.231 | 0.258 | 0.262 |
| 467 | 0.176 | 0.16 | 0.165 | 0.19 | 0.199 | 0.22 | 0.222 | 0.247 | 0.251 |
| 468 | 0.175 | 0.156 | 0.16 | 0.182 | 0.191 | 0.21 | 0.211 | 0.235 | 0.238 |
| 469 | 0.173 | 0.151 | 0.154 | 0.174 | 0.182 | 0.2 | 0.2 | 0.222 | 0.225 |
| 470 | 0.172 | 0.148 | 0.15 | 0.17 | 0.176 | 0.194 | 0.192 | 0.213 | 0.215 |
| 471 | 0.171 | 0.145 | 0.146 | 0.163 | 0.169 | 0.186 | 0.183 | 0.203 | 0.204 |
| 472 | 0.17 | 0.142 | 0.143 | 0.159 | 0.164 | 0.18 | 0.177 | 0.196 | 0.197 |
| 473 | 0.17 | 0.14 | 0.139 | 0.155 | 0.159 | 0.174 | 0.17 | 0.188 | 0.189 |
| 474 | 0.169 | 0.137 | 0.136 | 0.15 | 0.154 | 0.169 | 0.164 | 0.18 | 0.181 |
| 475 | 0.169 | 0.135 | 0.134 | 0.147 | 0.15 | 0.164 | 0.159 | 0.175 | 0.175 |
| 476 | 0.169 | 0.134 | 0.131 | 0.144 | 0.147 | 0.16 | 0.154 | 0.169 | 0.169 |
| 477 | 0.169 | 0.132 | 0.129 | 0.141 | 0.144 | 0.156 | 0.15 | 0.165 | 0.165 |
| 478 | 0.169 | 0.131 | 0.128 | 0.139 | 0.141 | 0.153 | 0.146 | 0.16 | 0.16 |
| 479 | 0.17 | 0.13 | 0.126 | 0.137 | 0.139 | 0.15 | 0.143 | 0.157 | 0.156 |
| 480 | 0.17 | 0.129 | 0.125 | 0.135 | 0.137 | 0.148 | 0.141 | 0.153 | 0.153 |
| 481 | 0.17 | 0.128 | 0.124 | 0.134 | 0.135 | 0.146 | 0.138 | 0.151 | 0.15 |
| 482 | 0.171 | 0.128 | 0.123 | 0.132 | 0.133 | 0.144 | 0.136 | 0.148 | 0.147 |
| 483 | 0.171 | 0.128 | 0.122 | 0.131 | 0.132 | 0.142 | 0.134 | 0.146 | 0.145 |
| 484 | 0.172 | 0.127 | 0.122 | 0.13 | 0.131 | 0.141 | 0.133 | 0.144 | 0.143 |
| 485 | 0.172 | 0.127 | 0.121 | 0.129 | 0.13 | 0.14 | 0.131 | 0.142 | 0.141 |
| 486 | 0.173 | 0.127 | 0.121 | 0.129 | 0.129 | 0.139 | 0.13 | 0.141 | 0.14 |
| 487 | 0.174 | 0.126 | 0.12 | 0.128 | 0.128 | 0.138 | 0.129 | 0.14 | 0.139 |
| 488 | 0.174 | 0.126 | 0.12 | 0.128 | 0.128 | 0.137 | 0.128 | 0.139 | 0.137 |
| 489 | 0.175 | 0.126 | 0.12 | 0.127 | 0.127 | 0.136 | 0.127 | 0.138 | 0.136 |
| 490 | 0.176 | 0.126 | 0.12 | 0.127 | 0.127 | 0.136 | 0.127 | 0.137 | 0.136 |
| 491 | 0.177 | 0.127 | 0.12 | 0.127 | 0.126 | 0.135 | 0.126 | 0.136 | 0.135 |
| 492 | 0.178 | 0.127 | 0.12 | 0.127 | 0.126 | 0.135 | 0.126 | 0.136 | 0.134 |
| 493 | 0.179 | 0.127 | 0.12 | 0.127 | 0.126 | 0.135 | 0.125 | 0.135 | 0.134 |
| 494 | 0.18 | 0.128 | 0.12 | 0.127 | 0.126 | 0.135 | 0.125 | 0.135 | 0.134 |
| 495 | 0.182 | 0.128 | 0.12 | 0.127 | 0.126 | 0.134 | 0.125 | 0.135 | 0.133 |
| 496 | 0.184 | 0.129 | 0.121 | 0.127 | 0.126 | 0.134 | 0.125 | 0.134 | 0.133 |
| 497 | 0.185 | 0.129 | 0.121 | 0.127 | 0.126 | 0.135 | 0.125 | 0.135 | 0.133 |
| 498 | 0.187 | 0.13 | 0.122 | 0.127 | 0.126 | 0.135 | 0.125 | 0.134 | 0.133 |
| 499 | 0.19 | 0.131 | 0.122 | 0.128 | 0.127 | 0.135 | 0.125 | 0.135 | 0.133 |

2-G

|  |  |  |  |
| --- | --- | --- | --- |
| 300 | 0.294 | 0.284 | 0.267 |
| 301 | 0.296 | 0.284 | 0.267 |
| 302 | 0.299 | 0.285 | 0.266 |
| 303 | 0.3 | 0.286 | 0.266 |
| 304 | 0.302 | 0.288 | 0.266 |
| 305 | 0.305 | 0.289 | 0.267 |
| 306 | 0.307 | 0.29 | 0.268 |
| 307 | 0.309 | 0.291 | 0.268 |
| 308 | 0.312 | 0.293 | 0.269 |
| 309 | 0.313 | 0.294 | 0.27 |
| 310 | 0.315 | 0.295 | 0.271 |
| 311 | 0.317 | 0.297 | 0.272 |
| 312 | 0.318 | 0.297 | 0.272 |
| 313 | 0.32 | 0.3 | 0.274 |
| 314 | 0.321 | 0.3 | 0.274 |
| 315 | 0.323 | 0.301 | 0.275 |
| 316 | 0.324 | 0.302 | 0.276 |
| 317 | 0.326 | 0.303 | 0.276 |
| 318 | 0.326 | 0.305 | 0.278 |
| 319 | 0.328 | 0.306 | 0.278 |
| 320 | 0.329 | 0.306 | 0.279 |
| 321 | 0.33 | 0.307 | 0.28 |
| 322 | 0.332 | 0.308 | 0.281 |
| 323 | 0.332 | 0.309 | 0.282 |
| 324 | 0.333 | 0.31 | 0.282 |
| 325 | 0.335 | 0.311 | 0.283 |
| 326 | 0.336 | 0.312 | 0.284 |
| 327 | 0.337 | 0.313 | 0.285 |
| 328 | 0.34 | 0.314 | 0.286 |
| 329 | 0.341 | 0.316 | 0.287 |
| 330 | 0.342 | 0.317 | 0.288 |
| 331 | 0.344 | 0.318 | 0.29 |
| 332 | 0.308 | 0.281 | 0.259 |
| 333 | 0.31 | 0.282 | 0.261 |
| 334 | 0.311 | 0.283 | 0.262 |
| 335 | 0.313 | 0.285 | 0.263 |
| 336 | 0.315 | 0.286 | 0.265 |
| 337 | 0.317 | 0.289 | 0.266 |
| 338 | 0.319 | 0.29 | 0.268 |
| 339 | 0.322 | 0.292 | 0.27 |
| 340 | 0.325 | 0.294 | 0.271 |
| 341 | 0.327 | 0.296 | 0.273 |
| 342 | 0.33 | 0.299 | 0.275 |
| 343 | 0.332 | 0.3 | 0.277 |
| 344 | 0.336 | 0.303 | 0.279 |
| 345 | 0.338 | 0.306 | 0.281 |
| 346 | 0.341 | 0.308 | 0.283 |
| 347 | 0.344 | 0.31 | 0.287 |
| 348 | 0.348 | 0.313 | 0.289 |
| 349 | 0.35 | 0.315 | 0.29 |
| 350 | 0.352 | 0.317 | 0.292 |
| 351 | 0.354 | 0.319 | 0.293 |
| 352 | 0.358 | 0.321 | 0.296 |
| 353 | 0.36 | 0.323 | 0.298 |
| 354 | 0.362 | 0.325 | 0.299 |
| 355 | 0.364 | 0.327 | 0.301 |
| 356 | 0.366 | 0.329 | 0.303 |
| 357 | 0.369 | 0.331 | 0.305 |
| 358 | 0.37 | 0.332 | 0.306 |
| 359 | 0.372 | 0.334 | 0.307 |
| 360 | 0.374 | 0.335 | 0.309 |
| 361 | 0.376 | 0.337 | 0.31 |
| 362 | 0.378 | 0.339 | 0.311 |
| 363 | 0.38 | 0.34 | 0.313 |
| 364 | 0.382 | 0.342 | 0.315 |
| 365 | 0.384 | 0.343 | 0.316 |
| 366 | 0.387 | 0.346 | 0.317 |
| 367 | 0.389 | 0.347 | 0.319 |
| 368 | 0.391 | 0.349 | 0.32 |
| 369 | 0.393 | 0.35 | 0.322 |
| 370 | 0.396 | 0.353 | 0.324 |
| 371 | 0.398 | 0.354 | 0.325 |
| 372 | 0.4 | 0.356 | 0.327 |
| 373 | 0.402 | 0.358 | 0.328 |
| 374 | 0.405 | 0.36 | 0.329 |
| 375 | 0.406 | 0.36 | 0.328 |
| 376 | 0.407 | 0.361 | 0.33 |
| 377 | 0.409 | 0.363 | 0.331 |
| 378 | 0.412 | 0.365 | 0.333 |
| 379 | 0.414 | 0.367 | 0.334 |
| 380 | 0.417 | 0.369 | 0.337 |
| 381 | 0.42 | 0.371 | 0.338 |
| 382 | 0.423 | 0.373 | 0.34 |
| 383 | 0.427 | 0.377 | 0.343 |
| 384 | 0.431 | 0.38 | 0.345 |
| 385 | 0.437 | 0.385 | 0.349 |
| 386 | 0.442 | 0.389 | 0.353 |
| 387 | 0.45 | 0.394 | 0.357 |
| 388 | 0.457 | 0.4 | 0.362 |
| 389 | 0.466 | 0.406 | 0.367 |
| 390 | 0.478 | 0.416 | 0.374 |
| 391 | 0.49 | 0.425 | 0.382 |
| 392 | 0.507 | 0.437 | 0.392 |
| 393 | 0.522 | 0.449 | 0.401 |
| 394 | 0.542 | 0.464 | 0.413 |
| 395 | 0.56 | 0.477 | 0.424 |
| 396 | 0.584 | 0.495 | 0.438 |
| 397 | 0.604 | 0.51 | 0.451 |
| 398 | 0.63 | 0.53 | 0.467 |
| 399 | 0.655 | 0.55 | 0.482 |
| 400 | 0.68 | 0.568 | 0.497 |
| 401 | 0.708 | 0.589 | 0.515 |
| 402 | 0.739 | 0.612 | 0.534 |
| 403 | 0.774 | 0.639 | 0.556 |
| 404 | 0.805 | 0.664 | 0.576 |
| 405 | 0.846 | 0.695 | 0.602 |
| 406 | 0.885 | 0.725 | 0.627 |
| 407 | 0.934 | 0.762 | 0.657 |
| 408 | 0.989 | 0.804 | 0.691 |
| 409 | 1.052 | 0.852 | 0.731 |
| 410 | 1.118 | 0.901 | 0.771 |
| 411 | 1.195 | 0.959 | 0.818 |
| 412 | 1.255 | 1.005 | 0.856 |
| 413 | 1.343 | 1.073 | 0.911 |
| 414 | 1.425 | 1.135 | 0.963 |
| 415 | 1.505 | 1.197 | 1.014 |
| 416 | 1.584 | 1.261 | 1.068 |
| 417 | 1.651 | 1.314 | 1.113 |
| 418 | 1.714 | 1.365 | 1.157 |
| 419 | 1.773 | 1.416 | 1.201 |
| 420 | 1.809 | 1.443 | 1.228 |
| 421 | 1.831 | 1.465 | 1.25 |
| 422 | 1.835 | 1.477 | 1.26 |
| 423 | 1.832 | 1.471 | 1.264 |
| 424 | 1.811 | 1.464 | 1.258 |
| 425 | 1.768 | 1.434 | 1.242 |
| 426 | 1.731 | 1.403 | 1.221 |
| 427 | 1.67 | 1.357 | 1.191 |
| 428 | 1.601 | 1.309 | 1.155 |
| 429 | 1.521 | 1.25 | 1.111 |
| 430 | 1.437 | 1.19 | 1.067 |
| 431 | 1.351 | 1.126 | 1.02 |
| 432 | 1.26 | 1.061 | 0.971 |
| 433 | 1.171 | 0.996 | 0.923 |
| 434 | 1.081 | 0.929 | 0.873 |
| 435 | 0.994 | 0.867 | 0.825 |
| 436 | 0.917 | 0.809 | 0.781 |
| 437 | 0.845 | 0.756 | 0.74 |
| 438 | 0.77 | 0.7 | 0.695 |
| 439 | 0.707 | 0.652 | 0.658 |
| 440 | 0.642 | 0.602 | 0.617 |
| 441 | 0.594 | 0.565 | 0.586 |
| 442 | 0.54 | 0.523 | 0.55 |
| 443 | 0.49 | 0.484 | 0.516 |
| 444 | 0.455 | 0.455 | 0.49 |
| 445 | 0.42 | 0.426 | 0.463 |
| 446 | 0.389 | 0.4 | 0.439 |
| 447 | 0.361 | 0.375 | 0.416 |
| 448 | 0.34 | 0.357 | 0.397 |
| 449 | 0.316 | 0.335 | 0.374 |
| 450 | 0.299 | 0.32 | 0.358 |
| 451 | 0.28 | 0.302 | 0.339 |
| 452 | 0.264 | 0.286 | 0.321 |
| 453 | 0.252 | 0.274 | 0.307 |
| 454 | 0.241 | 0.263 | 0.293 |
| 455 | 0.232 | 0.253 | 0.281 |
| 456 | 0.222 | 0.243 | 0.269 |
| 457 | 0.215 | 0.234 | 0.258 |
| 458 | 0.208 | 0.226 | 0.248 |
| 459 | 0.202 | 0.219 | 0.238 |
| 460 | 0.198 | 0.214 | 0.23 |
| 461 | 0.193 | 0.208 | 0.223 |
| 462 | 0.189 | 0.203 | 0.216 |
| 463 | 0.186 | 0.199 | 0.209 |
| 464 | 0.183 | 0.194 | 0.203 |
| 465 | 0.18 | 0.191 | 0.198 |
| 466 | 0.178 | 0.188 | 0.193 |
| 467 | 0.176 | 0.185 | 0.189 |
| 468 | 0.175 | 0.183 | 0.186 |
| 469 | 0.173 | 0.181 | 0.182 |
| 470 | 0.172 | 0.179 | 0.18 |
| 471 | 0.171 | 0.177 | 0.177 |
| 472 | 0.17 | 0.176 | 0.175 |
| 473 | 0.17 | 0.175 | 0.172 |
| 474 | 0.169 | 0.174 | 0.171 |
| 475 | 0.169 | 0.173 | 0.169 |
| 476 | 0.169 | 0.172 | 0.168 |
| 477 | 0.169 | 0.172 | 0.167 |
| 478 | 0.169 | 0.172 | 0.166 |
| 479 | 0.17 | 0.172 | 0.166 |
| 480 | 0.17 | 0.172 | 0.165 |
| 481 | 0.17 | 0.172 | 0.165 |
| 482 | 0.171 | 0.172 | 0.164 |
| 483 | 0.171 | 0.172 | 0.164 |
| 484 | 0.172 | 0.172 | 0.164 |
| 485 | 0.172 | 0.172 | 0.164 |
| 486 | 0.173 | 0.173 | 0.164 |
| 487 | 0.174 | 0.173 | 0.164 |
| 488 | 0.174 | 0.173 | 0.165 |
| 489 | 0.175 | 0.174 | 0.165 |
| 490 | 0.176 | 0.175 | 0.165 |
| 491 | 0.177 | 0.175 | 0.166 |
| 492 | 0.178 | 0.176 | 0.166 |
| 493 | 0.179 | 0.177 | 0.167 |
| 494 | 0.18 | 0.178 | 0.167 |
| 495 | 0.182 | 0.179 | 0.168 |
| 496 | 0.184 | 0.18 | 0.169 |
| 497 | 0.185 | 0.181 | 0.17 |
| 498 | 0.187 | 0.183 | 0.172 |
| 499 | 0.19 | 0.184 | 0.173 |

|  |  |  |  |  |  |
| --- | --- | --- | --- | --- | --- |
| 300 | 0.285 | 0.289 | 0.297 | 0.288 | 0.316 |
| 301 | 0.283 | 0.285 | 0.291 | 0.28 | 0.306 |
| 302 | 0.281 | 0.28 | 0.285 | 0.271 | 0.296 |
| 303 | 0.279 | 0.277 | 0.28 | 0.265 | 0.289 |
| 304 | 0.278 | 0.275 | 0.276 | 0.26 | 0.283 |
| 305 | 0.278 | 0.273 | 0.273 | 0.255 | 0.278 |
| 306 | 0.277 | 0.271 | 0.271 | 0.252 | 0.275 |
| 307 | 0.277 | 0.271 | 0.269 | 0.25 | 0.272 |
| 308 | 0.278 | 0.27 | 0.268 | 0.249 | 0.27 |
| 309 | 0.278 | 0.269 | 0.267 | 0.247 | 0.268 |
| 310 | 0.278 | 0.269 | 0.267 | 0.247 | 0.267 |
| 311 | 0.279 | 0.27 | 0.267 | 0.246 | 0.267 |
| 312 | 0.279 | 0.27 | 0.267 | 0.246 | 0.266 |
| 313 | 0.28 | 0.271 | 0.268 | 0.246 | 0.267 |
| 314 | 0.281 | 0.272 | 0.268 | 0.246 | 0.267 |
| 315 | 0.282 | 0.272 | 0.268 | 0.247 | 0.267 |
| 316 | 0.282 | 0.273 | 0.268 | 0.247 | 0.267 |
| 317 | 0.283 | 0.273 | 0.269 | 0.247 | 0.267 |
| 318 | 0.284 | 0.273 | 0.27 | 0.248 | 0.268 |
| 319 | 0.284 | 0.274 | 0.27 | 0.248 | 0.268 |
| 320 | 0.285 | 0.274 | 0.27 | 0.248 | 0.268 |
| 321 | 0.286 | 0.276 | 0.27 | 0.249 | 0.268 |
| 322 | 0.286 | 0.275 | 0.271 | 0.249 | 0.269 |
| 323 | 0.287 | 0.277 | 0.272 | 0.25 | 0.27 |
| 324 | 0.287 | 0.277 | 0.273 | 0.251 | 0.27 |
| 325 | 0.289 | 0.279 | 0.274 | 0.252 | 0.271 |
| 326 | 0.289 | 0.279 | 0.274 | 0.252 | 0.271 |
| 327 | 0.29 | 0.28 | 0.275 | 0.253 | 0.273 |
| 328 | 0.291 | 0.28 | 0.276 | 0.254 | 0.274 |
| 329 | 0.292 | 0.282 | 0.277 | 0.255 | 0.275 |
| 330 | 0.294 | 0.282 | 0.277 | 0.256 | 0.275 |
| 331 | 0.294 | 0.283 | 0.279 | 0.257 | 0.277 |
| 332 | 0.258 | 0.251 | 0.246 | 0.232 | 0.245 |
| 333 | 0.259 | 0.252 | 0.247 | 0.233 | 0.246 |
| 334 | 0.26 | 0.253 | 0.249 | 0.234 | 0.247 |
| 335 | 0.262 | 0.254 | 0.25 | 0.235 | 0.248 |
| 336 | 0.263 | 0.255 | 0.251 | 0.237 | 0.25 |
| 337 | 0.265 | 0.257 | 0.253 | 0.238 | 0.251 |
| 338 | 0.266 | 0.258 | 0.254 | 0.24 | 0.252 |
| 339 | 0.268 | 0.26 | 0.256 | 0.242 | 0.254 |
| 340 | 0.269 | 0.261 | 0.257 | 0.243 | 0.256 |
| 341 | 0.271 | 0.263 | 0.259 | 0.245 | 0.257 |
| 342 | 0.273 | 0.266 | 0.261 | 0.247 | 0.259 |
| 343 | 0.275 | 0.267 | 0.263 | 0.248 | 0.261 |
| 344 | 0.277 | 0.269 | 0.265 | 0.25 | 0.263 |
| 345 | 0.279 | 0.271 | 0.266 | 0.252 | 0.265 |
| 346 | 0.281 | 0.273 | 0.268 | 0.254 | 0.267 |
| 347 | 0.283 | 0.275 | 0.271 | 0.256 | 0.269 |
| 348 | 0.285 | 0.277 | 0.273 | 0.258 | 0.271 |
| 349 | 0.287 | 0.279 | 0.275 | 0.26 | 0.273 |
| 350 | 0.289 | 0.281 | 0.276 | 0.262 | 0.275 |
| 351 | 0.29 | 0.282 | 0.278 | 0.264 | 0.277 |
| 352 | 0.293 | 0.284 | 0.28 | 0.266 | 0.279 |
| 353 | 0.294 | 0.286 | 0.282 | 0.268 | 0.281 |
| 354 | 0.296 | 0.288 | 0.284 | 0.269 | 0.282 |
| 355 | 0.298 | 0.29 | 0.285 | 0.271 | 0.284 |
| 356 | 0.299 | 0.292 | 0.287 | 0.273 | 0.287 |
| 357 | 0.301 | 0.293 | 0.288 | 0.275 | 0.288 |
| 358 | 0.302 | 0.294 | 0.29 | 0.276 | 0.29 |
| 359 | 0.304 | 0.296 | 0.291 | 0.278 | 0.291 |
| 360 | 0.305 | 0.297 | 0.293 | 0.279 | 0.292 |
| 361 | 0.306 | 0.299 | 0.294 | 0.28 | 0.293 |
| 362 | 0.307 | 0.299 | 0.295 | 0.281 | 0.295 |
| 363 | 0.309 | 0.301 | 0.296 | 0.283 | 0.296 |
| 364 | 0.31 | 0.302 | 0.297 | 0.283 | 0.297 |
| 365 | 0.311 | 0.303 | 0.298 | 0.284 | 0.298 |
| 366 | 0.313 | 0.304 | 0.3 | 0.286 | 0.299 |
| 367 | 0.314 | 0.305 | 0.3 | 0.286 | 0.299 |
| 368 | 0.315 | 0.307 | 0.302 | 0.287 | 0.301 |
| 369 | 0.317 | 0.308 | 0.303 | 0.288 | 0.302 |
| 370 | 0.318 | 0.309 | 0.304 | 0.29 | 0.303 |
| 371 | 0.319 | 0.31 | 0.305 | 0.29 | 0.303 |
| 372 | 0.321 | 0.311 | 0.306 | 0.292 | 0.304 |
| 373 | 0.322 | 0.312 | 0.307 | 0.292 | 0.305 |
| 374 | 0.323 | 0.314 | 0.308 | 0.294 | 0.306 |
| 375 | 0.322 | 0.312 | 0.307 | 0.292 | 0.304 |
| 376 | 0.324 | 0.313 | 0.307 | 0.293 | 0.306 |
| 377 | 0.325 | 0.314 | 0.309 | 0.293 | 0.306 |
| 378 | 0.326 | 0.316 | 0.309 | 0.294 | 0.307 |
| 379 | 0.328 | 0.317 | 0.311 | 0.296 | 0.308 |
| 380 | 0.329 | 0.318 | 0.312 | 0.296 | 0.309 |
| 381 | 0.331 | 0.32 | 0.313 | 0.298 | 0.31 |
| 382 | 0.333 | 0.321 | 0.315 | 0.299 | 0.311 |
| 383 | 0.335 | 0.323 | 0.316 | 0.3 | 0.313 |
| 384 | 0.337 | 0.325 | 0.318 | 0.302 | 0.314 |
| 385 | 0.341 | 0.328 | 0.32 | 0.304 | 0.316 |
| 386 | 0.343 | 0.33 | 0.322 | 0.305 | 0.317 |
| 387 | 0.347 | 0.333 | 0.324 | 0.307 | 0.319 |
| 388 | 0.35 | 0.335 | 0.326 | 0.309 | 0.32 |
| 389 | 0.355 | 0.339 | 0.329 | 0.311 | 0.322 |
| 390 | 0.361 | 0.343 | 0.332 | 0.314 | 0.324 |
| 391 | 0.366 | 0.347 | 0.335 | 0.316 | 0.327 |
| 392 | 0.374 | 0.353 | 0.34 | 0.32 | 0.33 |
| 393 | 0.382 | 0.359 | 0.344 | 0.324 | 0.333 |
| 394 | 0.392 | 0.366 | 0.35 | 0.328 | 0.337 |
| 395 | 0.4 | 0.373 | 0.355 | 0.332 | 0.34 |
| 396 | 0.411 | 0.381 | 0.361 | 0.337 | 0.344 |
| 397 | 0.421 | 0.389 | 0.367 | 0.342 | 0.349 |
| 398 | 0.434 | 0.399 | 0.375 | 0.348 | 0.354 |
| 399 | 0.446 | 0.409 | 0.382 | 0.354 | 0.359 |
| 400 | 0.459 | 0.418 | 0.39 | 0.36 | 0.365 |
| 401 | 0.473 | 0.429 | 0.399 | 0.368 | 0.371 |
| 402 | 0.488 | 0.441 | 0.409 | 0.376 | 0.378 |
| 403 | 0.507 | 0.456 | 0.42 | 0.385 | 0.386 |
| 404 | 0.523 | 0.468 | 0.431 | 0.394 | 0.394 |
| 405 | 0.544 | 0.485 | 0.444 | 0.404 | 0.404 |
| 406 | 0.564 | 0.501 | 0.457 | 0.416 | 0.413 |
| 407 | 0.589 | 0.521 | 0.473 | 0.429 | 0.425 |
| 408 | 0.617 | 0.543 | 0.49 | 0.443 | 0.437 |
| 409 | 0.649 | 0.568 | 0.511 | 0.46 | 0.453 |
| 410 | 0.681 | 0.593 | 0.531 | 0.476 | 0.467 |
| 411 | 0.72 | 0.624 | 0.554 | 0.496 | 0.484 |
| 412 | 0.751 | 0.647 | 0.573 | 0.512 | 0.498 |
| 413 | 0.795 | 0.682 | 0.601 | 0.535 | 0.518 |
| 414 | 0.837 | 0.715 | 0.628 | 0.557 | 0.538 |
| 415 | 0.879 | 0.748 | 0.655 | 0.579 | 0.558 |
| 416 | 0.924 | 0.784 | 0.684 | 0.604 | 0.58 |
| 417 | 0.962 | 0.815 | 0.709 | 0.626 | 0.599 |
| 418 | 0.999 | 0.846 | 0.736 | 0.649 | 0.621 |
| 419 | 1.037 | 0.879 | 0.765 | 0.676 | 0.645 |
| 420 | 1.062 | 0.903 | 0.787 | 0.696 | 0.665 |
| 421 | 1.084 | 0.925 | 0.809 | 0.718 | 0.686 |
| 422 | 1.097 | 0.941 | 0.827 | 0.736 | 0.705 |
| 423 | 1.104 | 0.954 | 0.844 | 0.755 | 0.725 |
| 424 | 1.106 | 0.961 | 0.853 | 0.768 | 0.74 |
| 425 | 1.098 | 0.966 | 0.866 | 0.785 | 0.759 |
| 426 | 1.09 | 0.966 | 0.872 | 0.797 | 0.774 |
| 427 | 1.071 | 0.962 | 0.879 | 0.81 | 0.791 |
| 428 | 1.049 | 0.955 | 0.883 | 0.821 | 0.806 |
| 429 | 1.023 | 0.946 | 0.884 | 0.832 | 0.821 |
| 430 | 0.995 | 0.935 | 0.885 | 0.84 | 0.835 |
| 431 | 0.964 | 0.921 | 0.884 | 0.848 | 0.849 |
| 432 | 0.932 | 0.906 | 0.881 | 0.855 | 0.861 |
| 433 | 0.899 | 0.89 | 0.878 | 0.86 | 0.872 |
| 434 | 0.866 | 0.872 | 0.872 | 0.865 | 0.884 |
| 435 | 0.831 | 0.854 | 0.866 | 0.868 | 0.892 |
| 436 | 0.801 | 0.837 | 0.859 | 0.869 | 0.899 |
| 437 | 0.771 | 0.819 | 0.851 | 0.869 | 0.904 |
| 438 | 0.738 | 0.799 | 0.84 | 0.867 | 0.907 |
| 439 | 0.709 | 0.78 | 0.83 | 0.862 | 0.907 |
| 440 | 0.676 | 0.757 | 0.815 | 0.854 | 0.904 |
| 441 | 0.651 | 0.738 | 0.801 | 0.846 | 0.899 |
| 442 | 0.621 | 0.714 | 0.782 | 0.832 | 0.888 |
| 443 | 0.592 | 0.689 | 0.761 | 0.815 | 0.874 |
| 444 | 0.568 | 0.668 | 0.743 | 0.798 | 0.859 |
| 445 | 0.543 | 0.644 | 0.721 | 0.778 | 0.841 |
| 446 | 0.52 | 0.621 | 0.698 | 0.756 | 0.819 |
| 447 | 0.496 | 0.596 | 0.674 | 0.732 | 0.795 |
| 448 | 0.476 | 0.575 | 0.652 | 0.71 | 0.773 |
| 449 | 0.452 | 0.548 | 0.623 | 0.68 | 0.742 |
| 450 | 0.434 | 0.527 | 0.601 | 0.655 | 0.717 |
| 451 | 0.412 | 0.501 | 0.572 | 0.624 | 0.684 |
| 452 | 0.391 | 0.476 | 0.543 | 0.593 | 0.651 |
| 453 | 0.373 | 0.453 | 0.518 | 0.565 | 0.621 |
| 454 | 0.357 | 0.432 | 0.493 | 0.538 | 0.592 |
| 455 | 0.341 | 0.412 | 0.47 | 0.511 | 0.563 |
| 456 | 0.325 | 0.391 | 0.445 | 0.482 | 0.531 |
| 457 | 0.311 | 0.373 | 0.423 | 0.457 | 0.504 |
| 458 | 0.297 | 0.353 | 0.4 | 0.43 | 0.474 |
| 459 | 0.284 | 0.335 | 0.377 | 0.404 | 0.446 |
| 460 | 0.274 | 0.321 | 0.36 | 0.384 | 0.424 |
| 461 | 0.263 | 0.306 | 0.342 | 0.363 | 0.401 |
| 462 | 0.253 | 0.292 | 0.325 | 0.344 | 0.379 |
| 463 | 0.244 | 0.279 | 0.309 | 0.325 | 0.358 |
| 464 | 0.235 | 0.266 | 0.293 | 0.306 | 0.336 |
| 465 | 0.228 | 0.256 | 0.28 | 0.291 | 0.32 |
| 466 | 0.221 | 0.245 | 0.267 | 0.275 | 0.302 |
| 467 | 0.215 | 0.237 | 0.257 | 0.263 | 0.289 |
| 468 | 0.209 | 0.229 | 0.247 | 0.251 | 0.276 |
| 469 | 0.204 | 0.221 | 0.237 | 0.239 | 0.263 |
| 470 | 0.2 | 0.215 | 0.23 | 0.23 | 0.253 |
| 471 | 0.196 | 0.209 | 0.222 | 0.221 | 0.242 |
| 472 | 0.193 | 0.204 | 0.216 | 0.214 | 0.234 |
| 473 | 0.189 | 0.199 | 0.21 | 0.207 | 0.226 |
| 474 | 0.186 | 0.195 | 0.204 | 0.2 | 0.219 |
| 475 | 0.184 | 0.192 | 0.2 | 0.195 | 0.213 |
| 476 | 0.182 | 0.188 | 0.195 | 0.189 | 0.207 |
| 477 | 0.181 | 0.186 | 0.192 | 0.185 | 0.202 |
| 478 | 0.179 | 0.183 | 0.189 | 0.181 | 0.198 |
| 479 | 0.178 | 0.181 | 0.186 | 0.178 | 0.194 |
| 480 | 0.177 | 0.18 | 0.184 | 0.175 | 0.191 |
| 481 | 0.176 | 0.178 | 0.182 | 0.173 | 0.188 |
| 482 | 0.175 | 0.177 | 0.18 | 0.171 | 0.186 |
| 483 | 0.175 | 0.176 | 0.179 | 0.169 | 0.184 |
| 484 | 0.174 | 0.175 | 0.177 | 0.167 | 0.182 |
| 485 | 0.174 | 0.174 | 0.176 | 0.166 | 0.18 |
| 486 | 0.174 | 0.174 | 0.175 | 0.165 | 0.179 |
| 487 | 0.174 | 0.173 | 0.175 | 0.164 | 0.178 |
| 488 | 0.174 | 0.173 | 0.174 | 0.163 | 0.177 |
| 489 | 0.174 | 0.173 | 0.174 | 0.162 | 0.176 |
| 490 | 0.174 | 0.173 | 0.173 | 0.162 | 0.176 |
| 491 | 0.174 | 0.172 | 0.173 | 0.162 | 0.175 |
| 492 | 0.174 | 0.173 | 0.173 | 0.161 | 0.175 |
| 493 | 0.175 | 0.173 | 0.173 | 0.161 | 0.175 |
| 494 | 0.175 | 0.173 | 0.173 | 0.162 | 0.175 |
| 495 | 0.176 | 0.174 | 0.174 | 0.162 | 0.175 |
| 496 | 0.177 | 0.174 | 0.174 | 0.162 | 0.175 |
| 497 | 0.178 | 0.175 | 0.175 | 0.162 | 0.175 |
| 498 | 0.179 | 0.175 | 0.175 | 0.163 | 0.176 |

|  |  |
| --- | --- |
| 300 | 0.311 |
| 301 | 0.3 |
| 302 | 0.289 |
| 303 | 0.281 |
| 304 | 0.274 |
| 305 | 0.268 |
| 306 | 0.264 |
| 307 | 0.261 |
| 308 | 0.258 |
| 309 | 0.256 |
| 310 | 0.255 |
| 311 | 0.255 |
| 312 | 0.254 |
| 313 | 0.254 |
| 314 | 0.254 |
| 315 | 0.254 |
| 316 | 0.254 |
| 317 | 0.254 |
| 318 | 0.254 |
| 319 | 0.255 |
| 320 | 0.254 |
| 321 | 0.256 |
| 322 | 0.256 |
| 323 | 0.256 |
| 324 | 0.257 |
| 325 | 0.258 |
| 326 | 0.259 |
| 327 | 0.259 |
| 328 | 0.26 |
| 329 | 0.261 |
| 330 | 0.262 |
| 331 | 0.263 |
| 332 | 0.234 |
| 333 | 0.235 |
| 334 | 0.236 |
| 335 | 0.238 |
| 336 | 0.239 |
| 337 | 0.241 |
| 338 | 0.242 |
| 339 | 0.244 |
| 340 | 0.246 |
| 341 | 0.247 |
| 342 | 0.249 |
| 343 | 0.251 |
| 344 | 0.253 |
| 345 | 0.255 |
| 346 | 0.257 |
| 347 | 0.259 |
| 348 | 0.261 |
| 349 | 0.263 |
| 350 | 0.264 |
| 351 | 0.266 |
| 352 | 0.268 |
| 353 | 0.27 |
| 354 | 0.272 |
| 355 | 0.274 |
| 356 | 0.276 |
| 357 | 0.277 |
| 358 | 0.278 |
| 359 | 0.28 |
| 360 | 0.281 |
| 361 | 0.282 |
| 362 | 0.284 |
| 363 | 0.285 |
| 364 | 0.286 |
| 365 | 0.287 |
| 366 | 0.288 |
| 367 | 0.288 |
| 368 | 0.289 |
| 369 | 0.291 |
| 370 | 0.291 |
| 371 | 0.292 |
| 372 | 0.293 |
| 373 | 0.294 |
| 374 | 0.294 |
| 375 | 0.293 |
| 376 | 0.293 |
| 377 | 0.294 |
| 378 | 0.295 |
| 379 | 0.296 |
| 380 | 0.297 |
| 381 | 0.298 |
| 382 | 0.299 |
| 383 | 0.3 |
| 384 | 0.301 |
| 385 | 0.303 |
| 386 | 0.304 |
| 387 | 0.306 |
| 388 | 0.307 |
| 389 | 0.309 |
| 390 | 0.311 |
| 391 | 0.313 |
| 392 | 0.315 |
| 393 | 0.318 |
| 394 | 0.32 |
| 395 | 0.323 |
| 396 | 0.327 |
| 397 | 0.33 |
| 398 | 0.334 |
| 399 | 0.339 |
| 400 | 0.343 |
| 401 | 0.348 |
| 402 | 0.354 |
| 403 | 0.362 |
| 404 | 0.368 |
| 405 | 0.376 |
| 406 | 0.384 |
| 407 | 0.394 |
| 408 | 0.405 |
| 409 | 0.418 |
| 410 | 0.43 |
| 411 | 0.445 |
| 412 | 0.457 |
| 413 | 0.473 |
| 414 | 0.49 |
| 415 | 0.507 |
| 416 | 0.526 |
| 417 | 0.543 |
| 418 | 0.562 |
| 419 | 0.585 |
| 420 | 0.602 |
| 421 | 0.623 |
| 422 | 0.641 |
| 423 | 0.66 |
| 424 | 0.676 |
| 425 | 0.696 |
| 426 | 0.713 |
| 427 | 0.732 |
| 428 | 0.75 |
| 429 | 0.769 |
| 430 | 0.786 |
| 431 | 0.805 |
| 432 | 0.821 |
| 433 | 0.838 |
| 434 | 0.853 |
| 435 | 0.868 |
| 436 | 0.88 |
| 437 | 0.889 |
| 438 | 0.897 |
| 439 | 0.902 |
| 440 | 0.903 |
| 441 | 0.902 |
| 442 | 0.896 |
| 443 | 0.886 |
| 444 | 0.873 |
| 445 | 0.858 |
| 446 | 0.838 |
| 447 | 0.815 |
| 448 | 0.794 |
| 449 | 0.764 |
| 450 | 0.739 |
| 451 | 0.706 |
| 452 | 0.672 |
| 453 | 0.642 |
| 454 | 0.611 |
| 455 | 0.581 |
| 456 | 0.548 |
| 457 | 0.52 |
| 458 | 0.489 |
| 459 | 0.459 |
| 460 | 0.436 |
| 461 | 0.411 |
| 462 | 0.388 |
| 463 | 0.366 |
| 464 | 0.342 |
| 465 | 0.325 |
| 466 | 0.306 |
| 467 | 0.292 |
| 468 | 0.278 |
| 469 | 0.263 |
| 470 | 0.253 |
| 471 | 0.241 |
| 472 | 0.232 |
| 473 | 0.224 |
| 474 | 0.216 |
| 475 | 0.209 |
| 476 | 0.203 |
| 477 | 0.198 |
| 478 | 0.193 |
| 479 | 0.189 |
| 480 | 0.186 |
| 481 | 0.183 |
| 482 | 0.18 |
| 483 | 0.178 |
| 484 | 0.176 |
| 485 | 0.174 |
| 486 | 0.173 |
| 487 | 0.171 |
| 488 | 0.171 |
| 489 | 0.169 |
| 490 | 0.169 |
| 491 | 0.168 |
| 492 | 0.168 |
| 493 | 0.168 |
| 494 | 0.168 |
| 495 | 0.168 |
| 496 | 0.168 |
| 497 | 0.168 |
| 498 | 0.168 |
| 499 | 0.169 |

|  |  |  |  |
| --- | --- | --- | --- |
| 300 | 0.313 | 0.336 | 0.34 |
| 301 | 0.301 | 0.322 | 0.325 |
| 302 | 0.288 | 0.308 | 0.309 |
| 303 | 0.279 | 0.298 | 0.298 |
| 304 | 0.271 | 0.289 | 0.288 |
| 305 | 0.264 | 0.28 | 0.279 |
| 306 | 0.26 | 0.275 | 0.273 |
| 307 | 0.256 | 0.271 | 0.269 |
| 308 | 0.254 | 0.268 | 0.265 |
| 309 | 0.251 | 0.265 | 0.262 |
| 310 | 0.25 | 0.264 | 0.26 |
| 311 | 0.249 | 0.263 | 0.258 |
| 312 | 0.248 | 0.262 | 0.258 |
| 313 | 0.248 | 0.262 | 0.258 |
| 314 | 0.248 | 0.261 | 0.257 |
| 315 | 0.248 | 0.262 | 0.257 |
| 316 | 0.248 | 0.261 | 0.256 |
| 317 | 0.247 | 0.261 | 0.257 |
| 318 | 0.248 | 0.262 | 0.257 |
| 319 | 0.249 | 0.262 | 0.257 |
| 320 | 0.249 | 0.262 | 0.257 |
| 321 | 0.249 | 0.262 | 0.257 |
| 322 | 0.25 | 0.263 | 0.258 |
| 323 | 0.25 | 0.263 | 0.258 |
| 324 | 0.251 | 0.263 | 0.259 |
| 325 | 0.251 | 0.264 | 0.259 |
| 326 | 0.252 | 0.265 | 0.26 |
| 327 | 0.253 | 0.266 | 0.261 |
| 328 | 0.254 | 0.266 | 0.262 |
| 329 | 0.255 | 0.267 | 0.263 |
| 330 | 0.255 | 0.269 | 0.263 |
| 331 | 0.257 | 0.269 | 0.264 |
| 332 | 0.231 | 0.239 | 0.236 |
| 333 | 0.231 | 0.239 | 0.236 |
| 334 | 0.232 | 0.24 | 0.237 |
| 335 | 0.234 | 0.242 | 0.238 |
| 336 | 0.235 | 0.243 | 0.24 |
| 337 | 0.237 | 0.245 | 0.242 |
| 338 | 0.238 | 0.246 | 0.243 |
| 339 | 0.24 | 0.248 | 0.245 |
| 340 | 0.241 | 0.249 | 0.246 |
| 341 | 0.243 | 0.251 | 0.247 |
| 342 | 0.245 | 0.253 | 0.25 |
| 343 | 0.247 | 0.254 | 0.251 |
| 344 | 0.249 | 0.257 | 0.253 |
| 345 | 0.25 | 0.258 | 0.255 |
| 346 | 0.253 | 0.261 | 0.257 |
| 347 | 0.255 | 0.263 | 0.259 |
| 348 | 0.257 | 0.265 | 0.261 |
| 349 | 0.258 | 0.267 | 0.263 |
| 350 | 0.26 | 0.268 | 0.265 |
| 351 | 0.262 | 0.27 | 0.267 |
| 352 | 0.264 | 0.273 | 0.269 |
| 353 | 0.266 | 0.274 | 0.271 |
| 354 | 0.268 | 0.276 | 0.273 |
| 355 | 0.27 | 0.279 | 0.275 |
| 356 | 0.272 | 0.28 | 0.277 |
| 357 | 0.273 | 0.282 | 0.279 |
| 358 | 0.275 | 0.283 | 0.28 |
| 359 | 0.276 | 0.285 | 0.281 |
| 360 | 0.278 | 0.286 | 0.283 |
| 361 | 0.279 | 0.287 | 0.284 |
| 362 | 0.28 | 0.288 | 0.285 |
| 363 | 0.281 | 0.29 | 0.287 |
| 364 | 0.282 | 0.291 | 0.287 |
| 365 | 0.283 | 0.291 | 0.288 |
| 366 | 0.284 | 0.293 | 0.289 |
| 367 | 0.285 | 0.293 | 0.29 |
| 368 | 0.286 | 0.294 | 0.29 |
| 369 | 0.287 | 0.295 | 0.291 |
| 370 | 0.287 | 0.296 | 0.293 |
| 371 | 0.288 | 0.296 | 0.293 |
| 372 | 0.289 | 0.297 | 0.294 |
| 373 | 0.289 | 0.298 | 0.294 |
| 374 | 0.29 | 0.299 | 0.295 |
| 375 | 0.289 | 0.297 | 0.293 |
| 376 | 0.289 | 0.298 | 0.294 |
| 377 | 0.29 | 0.298 | 0.294 |
| 378 | 0.291 | 0.299 | 0.295 |
| 379 | 0.292 | 0.3 | 0.296 |
| 380 | 0.293 | 0.301 | 0.297 |
| 381 | 0.294 | 0.302 | 0.298 |
| 382 | 0.295 | 0.303 | 0.299 |
| 383 | 0.296 | 0.304 | 0.3 |
| 384 | 0.297 | 0.305 | 0.301 |
| 385 | 0.299 | 0.306 | 0.302 |
| 386 | 0.3 | 0.308 | 0.303 |
| 387 | 0.301 | 0.309 | 0.304 |
| 388 | 0.302 | 0.31 | 0.305 |
| 389 | 0.304 | 0.311 | 0.307 |
| 390 | 0.305 | 0.313 | 0.308 |
| 391 | 0.307 | 0.315 | 0.31 |
| 392 | 0.309 | 0.316 | 0.311 |
| 393 | 0.312 | 0.319 | 0.313 |
| 394 | 0.314 | 0.321 | 0.315 |
| 395 | 0.317 | 0.323 | 0.317 |
| 396 | 0.32 | 0.326 | 0.319 |
| 397 | 0.322 | 0.328 | 0.321 |
| 398 | 0.326 | 0.331 | 0.324 |
| 399 | 0.33 | 0.335 | 0.327 |
| 400 | 0.334 | 0.338 | 0.33 |
| 401 | 0.339 | 0.342 | 0.334 |
| 402 | 0.344 | 0.347 | 0.338 |
| 403 | 0.35 | 0.353 | 0.343 |
| 404 | 0.356 | 0.358 | 0.348 |
| 405 | 0.363 | 0.365 | 0.354 |
| 406 | 0.371 | 0.371 | 0.359 |
| 407 | 0.38 | 0.379 | 0.367 |
| 408 | 0.39 | 0.389 | 0.375 |
| 409 | 0.401 | 0.398 | 0.384 |
| 410 | 0.412 | 0.409 | 0.393 |
| 411 | 0.425 | 0.421 | 0.404 |
| 412 | 0.435 | 0.43 | 0.413 |
| 413 | 0.451 | 0.444 | 0.426 |
| 414 | 0.466 | 0.458 | 0.438 |
| 415 | 0.481 | 0.472 | 0.451 |
| 416 | 0.499 | 0.488 | 0.465 |
| 417 | 0.515 | 0.503 | 0.479 |
| 418 | 0.532 | 0.519 | 0.494 |
| 419 | 0.553 | 0.539 | 0.513 |
| 420 | 0.57 | 0.555 | 0.528 |
| 421 | 0.589 | 0.574 | 0.546 |
| 422 | 0.607 | 0.592 | 0.563 |
| 423 | 0.627 | 0.611 | 0.582 |
| 424 | 0.643 | 0.627 | 0.598 |
| 425 | 0.664 | 0.649 | 0.62 |
| 426 | 0.682 | 0.667 | 0.639 |
| 427 | 0.703 | 0.69 | 0.662 |
| 428 | 0.723 | 0.711 | 0.685 |
| 429 | 0.745 | 0.735 | 0.71 |
| 430 | 0.766 | 0.758 | 0.734 |
| 431 | 0.787 | 0.781 | 0.759 |
| 432 | 0.807 | 0.804 | 0.785 |
| 433 | 0.828 | 0.827 | 0.81 |
| 434 | 0.847 | 0.85 | 0.837 |
| 435 | 0.866 | 0.871 | 0.861 |
| 436 | 0.882 | 0.891 | 0.884 |
| 437 | 0.895 | 0.907 | 0.903 |
| 438 | 0.908 | 0.923 | 0.923 |
| 439 | 0.916 | 0.935 | 0.938 |
| 440 | 0.922 | 0.944 | 0.951 |
| 441 | 0.923 | 0.948 | 0.958 |
| 442 | 0.921 | 0.948 | 0.961 |
| 443 | 0.912 | 0.943 | 0.959 |
| 444 | 0.903 | 0.935 | 0.953 |
| 445 | 0.888 | 0.921 | 0.942 |
| 446 | 0.87 | 0.905 | 0.927 |
| 447 | 0.848 | 0.884 | 0.907 |
| 448 | 0.827 | 0.863 | 0.887 |
| 449 | 0.797 | 0.833 | 0.858 |
| 450 | 0.772 | 0.808 | 0.833 |
| 451 | 0.738 | 0.775 | 0.798 |
| 452 | 0.703 | 0.739 | 0.762 |
| 453 | 0.671 | 0.706 | 0.729 |
| 454 | 0.639 | 0.673 | 0.695 |
| 455 | 0.608 | 0.641 | 0.662 |
| 456 | 0.573 | 0.604 | 0.624 |
| 457 | 0.543 | 0.573 | 0.592 |
| 458 | 0.51 | 0.539 | 0.556 |
| 459 | 0.478 | 0.505 | 0.52 |
| 460 | 0.453 | 0.479 | 0.493 |
| 461 | 0.427 | 0.451 | 0.464 |
| 462 | 0.402 | 0.425 | 0.436 |
| 463 | 0.378 | 0.4 | 0.41 |
| 464 | 0.353 | 0.374 | 0.383 |
| 465 | 0.334 | 0.354 | 0.361 |
| 466 | 0.314 | 0.332 | 0.339 |
| 467 | 0.299 | 0.317 | 0.322 |
| 468 | 0.283 | 0.3 | 0.305 |
| 469 | 0.268 | 0.284 | 0.287 |
| 470 | 0.257 | 0.272 | 0.275 |
| 471 | 0.244 | 0.258 | 0.26 |
| 472 | 0.235 | 0.249 | 0.25 |
| 473 | 0.226 | 0.239 | 0.24 |
| 474 | 0.217 | 0.23 | 0.23 |
| 475 | 0.21 | 0.222 | 0.222 |
| 476 | 0.203 | 0.215 | 0.214 |
| 477 | 0.197 | 0.209 | 0.208 |
| 478 | 0.192 | 0.203 | 0.202 |
| 479 | 0.188 | 0.199 | 0.197 |
| 480 | 0.185 | 0.195 | 0.194 |
| 481 | 0.181 | 0.192 | 0.19 |
| 482 | 0.178 | 0.188 | 0.186 |
| 483 | 0.175 | 0.186 | 0.183 |
| 484 | 0.173 | 0.183 | 0.181 |
| 485 | 0.171 | 0.181 | 0.179 |
| 486 | 0.17 | 0.18 | 0.177 |
| 487 | 0.168 | 0.178 | 0.175 |
| 488 | 0.168 | 0.177 | 0.174 |
| 489 | 0.167 | 0.176 | 0.173 |
| 490 | 0.165 | 0.175 | 0.172 |
| 491 | 0.165 | 0.174 | 0.171 |
| 492 | 0.164 | 0.174 | 0.17 |
| 493 | 0.164 | 0.173 | 0.17 |
| 494 | 0.164 | 0.173 | 0.17 |
| 495 | 0.164 | 0.173 | 0.17 |
| 496 | 0.164 | 0.173 | 0.17 |
| 497 | 0.164 | 0.173 | 0.17 |
| 498 | 0.164 | 0.173 | 0.17 |

|  |  |
| --- | --- |
| 300 | 0.353 |
| 301 | 0.336 |
| 302 | 0.319 |
| 303 | 0.307 |
| 304 | 0.295 |
| 305 | 0.285 |
| 306 | 0.279 |
| 307 | 0.274 |
| 308 | 0.27 |
| 309 | 0.266 |
| 310 | 0.264 |
| 311 | 0.263 |
| 312 | 0.261 |
| 313 | 0.261 |
| 314 | 0.26 |
| 315 | 0.26 |
| 316 | 0.26 |
| 317 | 0.26 |
| 318 | 0.26 |
| 319 | 0.26 |
| 320 | 0.26 |
| 321 | 0.261 |
| 322 | 0.26 |
| 323 | 0.261 |
| 324 | 0.262 |
| 325 | 0.262 |
| 326 | 0.263 |
| 327 | 0.263 |
| 328 | 0.264 |
| 329 | 0.265 |
| 330 | 0.266 |
| 331 | 0.267 |
| 332 | 0.238 |
| 333 | 0.239 |
| 334 | 0.239 |
| 335 | 0.241 |
| 336 | 0.242 |
| 337 | 0.244 |
| 338 | 0.246 |
| 339 | 0.247 |
| 340 | 0.249 |
| 341 | 0.25 |
| 342 | 0.252 |
| 343 | 0.254 |
| 344 | 0.256 |
| 345 | 0.258 |
| 346 | 0.26 |
| 347 | 0.262 |
| 348 | 0.264 |
| 349 | 0.266 |
| 350 | 0.268 |
| 351 | 0.27 |
| 352 | 0.272 |
| 353 | 0.274 |
| 354 | 0.276 |
| 355 | 0.278 |
| 356 | 0.28 |
| 357 | 0.281 |
| 358 | 0.283 |
| 359 | 0.285 |
| 360 | 0.286 |
| 361 | 0.287 |
| 362 | 0.288 |
| 363 | 0.289 |
| 364 | 0.291 |
| 365 | 0.291 |
| 366 | 0.292 |
| 367 | 0.293 |
| 368 | 0.294 |
| 369 | 0.295 |
| 370 | 0.295 |
| 371 | 0.296 |
| 372 | 0.297 |
| 373 | 0.297 |
| 374 | 0.298 |
| 375 | 0.296 |
| 376 | 0.297 |
| 377 | 0.297 |
| 378 | 0.298 |
| 379 | 0.299 |
| 380 | 0.3 |
| 381 | 0.301 |
| 382 | 0.302 |
| 383 | 0.303 |
| 384 | 0.304 |
| 385 | 0.305 |
| 386 | 0.306 |
| 387 | 0.307 |
| 388 | 0.308 |
| 389 | 0.31 |
| 390 | 0.311 |
| 391 | 0.312 |
| 392 | 0.313 |
| 393 | 0.315 |
| 394 | 0.317 |
| 395 | 0.319 |
| 396 | 0.321 |
| 397 | 0.323 |
| 398 | 0.325 |
| 399 | 0.328 |
| 400 | 0.331 |
| 401 | 0.334 |
| 402 | 0.338 |
| 403 | 0.343 |
| 404 | 0.347 |
| 405 | 0.353 |
| 406 | 0.358 |
| 407 | 0.365 |
| 408 | 0.373 |
| 409 | 0.382 |
| 410 | 0.39 |
| 411 | 0.4 |
| 412 | 0.408 |
| 413 | 0.42 |
| 414 | 0.432 |
| 415 | 0.444 |
| 416 | 0.458 |
| 417 | 0.471 |
| 418 | 0.486 |
| 419 | 0.503 |
| 420 | 0.518 |
| 421 | 0.536 |
| 422 | 0.552 |
| 423 | 0.571 |
| 424 | 0.587 |
| 425 | 0.609 |
| 426 | 0.628 |
| 427 | 0.653 |
| 428 | 0.675 |
| 429 | 0.702 |
| 430 | 0.727 |
| 431 | 0.754 |
| 432 | 0.781 |
| 433 | 0.808 |
| 434 | 0.837 |
| 435 | 0.864 |
| 436 | 0.889 |
| 437 | 0.911 |
| 438 | 0.933 |
| 439 | 0.95 |
| 440 | 0.966 |
| 441 | 0.975 |
| 442 | 0.98 |
| 443 | 0.981 |
| 444 | 0.976 |
| 445 | 0.967 |
| 446 | 0.953 |
| 447 | 0.934 |
| 448 | 0.914 |
| 449 | 0.886 |
| 450 | 0.86 |
| 451 | 0.826 |
| 452 | 0.789 |
| 453 | 0.756 |
| 454 | 0.721 |
| 455 | 0.686 |
| 456 | 0.647 |
| 457 | 0.613 |
| 458 | 0.576 |
| 459 | 0.54 |
| 460 | 0.512 |
| 461 | 0.481 |
| 462 | 0.452 |
| 463 | 0.425 |
| 464 | 0.396 |
| 465 | 0.374 |
| 466 | 0.35 |
| 467 | 0.332 |
| 468 | 0.314 |
| 469 | 0.296 |
| 470 | 0.283 |
| 471 | 0.268 |
| 472 | 0.257 |
| 473 | 0.246 |
| 474 | 0.236 |
| 475 | 0.228 |
| 476 | 0.219 |
| 477 | 0.213 |
| 478 | 0.207 |
| 479 | 0.202 |
| 480 | 0.198 |
| 481 | 0.193 |
| 482 | 0.189 |
| 483 | 0.187 |
| 484 | 0.184 |
| 485 | 0.182 |
| 486 | 0.18 |
| 487 | 0.178 |
| 488 | 0.177 |
| 489 | 0.176 |
| 490 | 0.174 |
| 491 | 0.174 |
| 492 | 0.173 |
| 493 | 0.173 |
| 494 | 0.172 |
| 495 | 0.172 |
| 496 | 0.172 |
| 497 | 0.172 |
| 498 | 0.172 |
| 499 | 0.173 |

|  |  |  |
| --- | --- | --- |
| 300 | 0.353 | 0.351 |
| 301 | 0.335 | 0.332 |
| 302 | 0.315 | 0.309 |
| 303 | 0.301 | 0.294 |
| 304 | 0.288 | 0.28 |
| 305 | 0.277 | 0.267 |
| 306 | 0.27 | 0.259 |
| 307 | 0.264 | 0.253 |
| 308 | 0.259 | 0.247 |
| 309 | 0.256 | 0.243 |
| 310 | 0.253 | 0.24 |
| 311 | 0.251 | 0.238 |
| 312 | 0.25 | 0.236 |
| 313 | 0.249 | 0.236 |
| 314 | 0.248 | 0.235 |
| 315 | 0.248 | 0.235 |
| 316 | 0.248 | 0.235 |
| 317 | 0.248 | 0.234 |
| 318 | 0.248 | 0.234 |
| 319 | 0.248 | 0.234 |
| 320 | 0.247 | 0.234 |
| 321 | 0.248 | 0.234 |
| 322 | 0.248 | 0.234 |
| 323 | 0.248 | 0.235 |
| 324 | 0.249 | 0.235 |
| 325 | 0.25 | 0.236 |
| 326 | 0.25 | 0.236 |
| 327 | 0.25 | 0.237 |
| 328 | 0.252 | 0.238 |
| 329 | 0.252 | 0.239 |
| 330 | 0.253 | 0.24 |
| 331 | 0.254 | 0.24 |
| 332 | 0.231 | 0.22 |
| 333 | 0.232 | 0.221 |
| 334 | 0.233 | 0.222 |
| 335 | 0.234 | 0.224 |
| 336 | 0.235 | 0.225 |
| 337 | 0.237 | 0.227 |
| 338 | 0.238 | 0.228 |
| 339 | 0.24 | 0.23 |
| 340 | 0.241 | 0.231 |
| 341 | 0.243 | 0.233 |
| 342 | 0.245 | 0.235 |
| 343 | 0.247 | 0.237 |
| 344 | 0.249 | 0.239 |
| 345 | 0.25 | 0.241 |
| 346 | 0.252 | 0.243 |
| 347 | 0.255 | 0.245 |
| 348 | 0.257 | 0.247 |
| 349 | 0.259 | 0.249 |
| 350 | 0.261 | 0.25 |
| 351 | 0.262 | 0.252 |
| 352 | 0.265 | 0.255 |
| 353 | 0.266 | 0.257 |
| 354 | 0.268 | 0.259 |
| 355 | 0.27 | 0.261 |
| 356 | 0.272 | 0.263 |
| 357 | 0.274 | 0.264 |
| 358 | 0.276 | 0.266 |
| 359 | 0.277 | 0.268 |
| 360 | 0.279 | 0.269 |
| 361 | 0.28 | 0.27 |
| 362 | 0.281 | 0.271 |
| 363 | 0.282 | 0.273 |
| 364 | 0.283 | 0.274 |
| 365 | 0.284 | 0.274 |
| 366 | 0.285 | 0.275 |
| 367 | 0.285 | 0.276 |
| 368 | 0.286 | 0.277 |
| 369 | 0.287 | 0.278 |
| 370 | 0.288 | 0.278 |
| 371 | 0.288 | 0.279 |
| 372 | 0.289 | 0.28 |
| 373 | 0.29 | 0.28 |
| 374 | 0.29 | 0.281 |
| 375 | 0.288 | 0.279 |
| 376 | 0.289 | 0.28 |
| 377 | 0.289 | 0.281 |
| 378 | 0.29 | 0.281 |
| 379 | 0.291 | 0.282 |
| 380 | 0.292 | 0.283 |
| 381 | 0.293 | 0.284 |
| 382 | 0.294 | 0.284 |
| 383 | 0.295 | 0.286 |
| 384 | 0.296 | 0.287 |
| 385 | 0.297 | 0.288 |
| 386 | 0.298 | 0.289 |
| 387 | 0.299 | 0.29 |
| 388 | 0.299 | 0.29 |
| 389 | 0.301 | 0.292 |
| 390 | 0.302 | 0.293 |
| 391 | 0.304 | 0.294 |
| 392 | 0.305 | 0.296 |
| 393 | 0.306 | 0.297 |
| 394 | 0.308 | 0.299 |
| 395 | 0.31 | 0.3 |
| 396 | 0.311 | 0.302 |
| 397 | 0.313 | 0.303 |
| 398 | 0.316 | 0.306 |
| 399 | 0.318 | 0.308 |
| 400 | 0.32 | 0.31 |
| 401 | 0.324 | 0.313 |
| 402 | 0.327 | 0.316 |
| 403 | 0.332 | 0.321 |
| 404 | 0.336 | 0.324 |
| 405 | 0.341 | 0.33 |
| 406 | 0.346 | 0.334 |
| 407 | 0.352 | 0.34 |
| 408 | 0.36 | 0.347 |
| 409 | 0.368 | 0.355 |
| 410 | 0.376 | 0.363 |
| 411 | 0.385 | 0.372 |
| 412 | 0.393 | 0.379 |
| 413 | 0.404 | 0.39 |
| 414 | 0.415 | 0.401 |
| 415 | 0.427 | 0.412 |
| 416 | 0.44 | 0.424 |
| 417 | 0.452 | 0.436 |
| 418 | 0.466 | 0.449 |
| 419 | 0.483 | 0.466 |
| 420 | 0.497 | 0.479 |
| 421 | 0.514 | 0.496 |
| 422 | 0.53 | 0.511 |
| 423 | 0.549 | 0.53 |
| 424 | 0.564 | 0.545 |
| 425 | 0.586 | 0.567 |
| 426 | 0.605 | 0.586 |
| 427 | 0.629 | 0.61 |
| 428 | 0.652 | 0.634 |
| 429 | 0.679 | 0.661 |
| 430 | 0.706 | 0.688 |
| 431 | 0.733 | 0.716 |
| 432 | 0.762 | 0.746 |
| 433 | 0.791 | 0.776 |
| 434 | 0.821 | 0.808 |
| 435 | 0.85 | 0.838 |
| 436 | 0.877 | 0.867 |
| 437 | 0.901 | 0.893 |
| 438 | 0.926 | 0.921 |
| 439 | 0.946 | 0.941 |
| 440 | 0.965 | 0.962 |
| 441 | 0.976 | 0.975 |
| 442 | 0.984 | 0.986 |
| 443 | 0.987 | 0.99 |
| 444 | 0.984 | 0.989 |
| 445 | 0.977 | 0.984 |
| 446 | 0.964 | 0.972 |
| 447 | 0.946 | 0.955 |
| 448 | 0.927 | 0.937 |
| 449 | 0.9 | 0.909 |
| 450 | 0.875 | 0.885 |
| 451 | 0.84 | 0.85 |
| 452 | 0.803 | 0.812 |
| 453 | 0.769 | 0.778 |
| 454 | 0.733 | 0.742 |
| 455 | 0.698 | 0.706 |
| 456 | 0.658 | 0.665 |
| 457 | 0.623 | 0.629 |
| 458 | 0.585 | 0.59 |
| 459 | 0.547 | 0.551 |
| 460 | 0.518 | 0.521 |
| 461 | 0.486 | 0.488 |
| 462 | 0.456 | 0.458 |
| 463 | 0.428 | 0.428 |
| 464 | 0.398 | 0.397 |
| 465 | 0.374 | 0.373 |
| 466 | 0.349 | 0.347 |
| 467 | 0.331 | 0.329 |
| 468 | 0.312 | 0.309 |
| 469 | 0.293 | 0.289 |
| 470 | 0.28 | 0.275 |
| 471 | 0.264 | 0.259 |
| 472 | 0.253 | 0.247 |
| 473 | 0.241 | 0.236 |
| 474 | 0.23 | 0.224 |
| 475 | 0.222 | 0.216 |
| 476 | 0.213 | 0.207 |
| 477 | 0.207 | 0.2 |
| 478 | 0.2 | 0.193 |
| 479 | 0.195 | 0.188 |
| 480 | 0.19 | 0.183 |
| 481 | 0.186 | 0.179 |
| 482 | 0.182 | 0.174 |
| 483 | 0.179 | 0.172 |
| 484 | 0.176 | 0.169 |
| 485 | 0.174 | 0.166 |
| 486 | 0.172 | 0.164 |
| 487 | 0.17 | 0.162 |
| 488 | 0.169 | 0.161 |
| 489 | 0.167 | 0.16 |
| 490 | 0.166 | 0.158 |
| 491 | 0.165 | 0.157 |
| 492 | 0.164 | 0.157 |
| 493 | 0.164 | 0.156 |
| 494 | 0.164 | 0.156 |
| 495 | 0.164 | 0.155 |
| 496 | 0.163 | 0.155 |
| 497 | 0.164 | 0.155 |
| 498 | 0.164 | 0.155 |

3-G

|  |  |  |  |  |  |  |  |  |  |  |
| --- | --- | --- | --- | --- | --- | --- | --- | --- | --- | --- |
| 300 | 0.303 | 0.328 | 0.325 | 0.328 | 0.345 | 0.337 | 0.364 | 0.372 | 0.408 | 0.439 |
| 301 | 0.306 | 0.328 | 0.323 | 0.324 | 0.338 | 0.328 | 0.352 | 0.357 | 0.392 | 0.419 |
| 302 | 0.308 | 0.328 | 0.322 | 0.32 | 0.332 | 0.318 | 0.339 | 0.342 | 0.375 | 0.399 |
| 303 | 0.31 | 0.329 | 0.321 | 0.318 | 0.327 | 0.311 | 0.331 | 0.331 | 0.363 | 0.385 |
| 304 | 0.312 | 0.329 | 0.32 | 0.315 | 0.323 | 0.305 | 0.322 | 0.322 | 0.352 | 0.371 |
| 305 | 0.315 | 0.331 | 0.32 | 0.314 | 0.319 | 0.3 | 0.316 | 0.314 | 0.342 | 0.36 |
| 306 | 0.317 | 0.332 | 0.32 | 0.313 | 0.317 | 0.297 | 0.311 | 0.308 | 0.336 | 0.352 |
| 307 | 0.319 | 0.333 | 0.321 | 0.312 | 0.316 | 0.294 | 0.308 | 0.305 | 0.332 | 0.346 |
| 308 | 0.322 | 0.334 | 0.321 | 0.312 | 0.315 | 0.293 | 0.305 | 0.301 | 0.328 | 0.342 |
| 309 | 0.323 | 0.335 | 0.322 | 0.312 | 0.314 | 0.291 | 0.304 | 0.299 | 0.325 | 0.338 |
| 310 | 0.326 | 0.336 | 0.323 | 0.313 | 0.314 | 0.291 | 0.302 | 0.298 | 0.323 | 0.335 |
| 311 | 0.328 | 0.338 | 0.323 | 0.313 | 0.314 | 0.291 | 0.302 | 0.297 | 0.322 | 0.334 |
| 312 | 0.329 | 0.339 | 0.324 | 0.313 | 0.314 | 0.29 | 0.301 | 0.296 | 0.321 | 0.332 |
| 313 | 0.331 | 0.34 | 0.326 | 0.314 | 0.315 | 0.29 | 0.302 | 0.296 | 0.321 | 0.333 |
| 314 | 0.332 | 0.341 | 0.326 | 0.315 | 0.315 | 0.29 | 0.301 | 0.296 | 0.321 | 0.332 |
| 315 | 0.334 | 0.342 | 0.328 | 0.316 | 0.315 | 0.291 | 0.302 | 0.296 | 0.321 | 0.331 |
| 316 | 0.336 | 0.343 | 0.328 | 0.316 | 0.316 | 0.291 | 0.302 | 0.296 | 0.321 | 0.331 |
| 317 | 0.337 | 0.344 | 0.329 | 0.317 | 0.316 | 0.291 | 0.302 | 0.296 | 0.321 | 0.331 |
| 318 | 0.338 | 0.345 | 0.33 | 0.318 | 0.317 | 0.292 | 0.303 | 0.296 | 0.321 | 0.331 |
| 319 | 0.339 | 0.346 | 0.33 | 0.318 | 0.318 | 0.293 | 0.302 | 0.297 | 0.321 | 0.332 |
| 320 | 0.341 | 0.347 | 0.331 | 0.319 | 0.318 | 0.293 | 0.303 | 0.297 | 0.321 | 0.332 |
| 321 | 0.342 | 0.348 | 0.332 | 0.319 | 0.319 | 0.293 | 0.304 | 0.298 | 0.322 | 0.332 |
| 322 | 0.343 | 0.349 | 0.333 | 0.32 | 0.319 | 0.294 | 0.304 | 0.299 | 0.322 | 0.333 |
| 323 | 0.344 | 0.35 | 0.333 | 0.32 | 0.32 | 0.295 | 0.305 | 0.299 | 0.323 | 0.333 |
| 324 | 0.346 | 0.351 | 0.335 | 0.322 | 0.321 | 0.295 | 0.306 | 0.3 | 0.323 | 0.334 |
| 325 | 0.348 | 0.352 | 0.336 | 0.323 | 0.322 | 0.296 | 0.307 | 0.301 | 0.324 | 0.334 |
| 326 | 0.348 | 0.353 | 0.336 | 0.323 | 0.322 | 0.297 | 0.307 | 0.301 | 0.325 | 0.334 |
| 327 | 0.35 | 0.354 | 0.337 | 0.324 | 0.323 | 0.298 | 0.308 | 0.302 | 0.326 | 0.335 |
| 328 | 0.352 | 0.355 | 0.338 | 0.326 | 0.324 | 0.299 | 0.309 | 0.304 | 0.327 | 0.337 |
| 329 | 0.353 | 0.357 | 0.34 | 0.327 | 0.325 | 0.3 | 0.31 | 0.305 | 0.328 | 0.337 |
| 330 | 0.355 | 0.357 | 0.341 | 0.327 | 0.327 | 0.301 | 0.31 | 0.305 | 0.328 | 0.338 |
| 331 | 0.356 | 0.36 | 0.342 | 0.329 | 0.327 | 0.302 | 0.312 | 0.307 | 0.33 | 0.339 |
| 332 | 0.313 | 0.298 | 0.281 | 0.272 | 0.267 | 0.257 | 0.262 | 0.254 | 0.275 | 0.283 |
| 333 | 0.315 | 0.299 | 0.282 | 0.273 | 0.268 | 0.258 | 0.263 | 0.255 | 0.277 | 0.285 |
| 334 | 0.317 | 0.3 | 0.283 | 0.273 | 0.269 | 0.259 | 0.264 | 0.256 | 0.277 | 0.286 |
| 335 | 0.319 | 0.302 | 0.285 | 0.275 | 0.271 | 0.26 | 0.265 | 0.258 | 0.279 | 0.287 |
| 336 | 0.321 | 0.304 | 0.286 | 0.276 | 0.272 | 0.262 | 0.267 | 0.26 | 0.28 | 0.289 |
| 337 | 0.323 | 0.306 | 0.289 | 0.278 | 0.274 | 0.263 | 0.268 | 0.262 | 0.282 | 0.29 |
| 338 | 0.325 | 0.308 | 0.29 | 0.28 | 0.275 | 0.265 | 0.27 | 0.263 | 0.283 | 0.292 |
| 339 | 0.328 | 0.31 | 0.292 | 0.282 | 0.277 | 0.267 | 0.272 | 0.265 | 0.286 | 0.294 |
| 340 | 0.331 | 0.311 | 0.294 | 0.284 | 0.279 | 0.268 | 0.273 | 0.266 | 0.287 | 0.295 |
| 341 | 0.333 | 0.313 | 0.295 | 0.285 | 0.281 | 0.27 | 0.275 | 0.268 | 0.289 | 0.297 |
| 342 | 0.337 | 0.316 | 0.298 | 0.287 | 0.282 | 0.272 | 0.277 | 0.271 | 0.291 | 0.299 |
| 343 | 0.34 | 0.318 | 0.3 | 0.289 | 0.284 | 0.274 | 0.279 | 0.272 | 0.293 | 0.301 |
| 344 | 0.343 | 0.321 | 0.302 | 0.291 | 0.287 | 0.276 | 0.281 | 0.275 | 0.295 | 0.303 |
| 345 | 0.346 | 0.323 | 0.304 | 0.293 | 0.289 | 0.278 | 0.283 | 0.276 | 0.297 | 0.305 |
| 346 | 0.349 | 0.326 | 0.307 | 0.295 | 0.291 | 0.28 | 0.285 | 0.278 | 0.299 | 0.307 |
| 347 | 0.353 | 0.329 | 0.309 | 0.298 | 0.293 | 0.283 | 0.287 | 0.281 | 0.302 | 0.309 |
| 348 | 0.355 | 0.331 | 0.311 | 0.3 | 0.295 | 0.285 | 0.289 | 0.283 | 0.304 | 0.312 |
| 349 | 0.359 | 0.333 | 0.313 | 0.302 | 0.297 | 0.287 | 0.291 | 0.286 | 0.306 | 0.314 |
| 350 | 0.361 | 0.335 | 0.315 | 0.304 | 0.299 | 0.289 | 0.293 | 0.288 | 0.307 | 0.316 |
| 351 | 0.363 | 0.337 | 0.317 | 0.306 | 0.301 | 0.29 | 0.295 | 0.289 | 0.309 | 0.317 |
| 352 | 0.366 | 0.34 | 0.319 | 0.308 | 0.303 | 0.293 | 0.298 | 0.292 | 0.312 | 0.32 |
| 353 | 0.369 | 0.341 | 0.321 | 0.31 | 0.305 | 0.295 | 0.299 | 0.294 | 0.314 | 0.322 |
| 354 | 0.371 | 0.343 | 0.322 | 0.311 | 0.306 | 0.296 | 0.301 | 0.295 | 0.316 | 0.324 |
| 355 | 0.374 | 0.345 | 0.325 | 0.313 | 0.308 | 0.299 | 0.303 | 0.298 | 0.318 | 0.326 |
| 356 | 0.376 | 0.347 | 0.327 | 0.315 | 0.31 | 0.3 | 0.305 | 0.3 | 0.32 | 0.328 |
| 357 | 0.378 | 0.349 | 0.328 | 0.317 | 0.312 | 0.302 | 0.307 | 0.301 | 0.322 | 0.33 |
| 358 | 0.38 | 0.35 | 0.329 | 0.318 | 0.313 | 0.304 | 0.308 | 0.303 | 0.323 | 0.332 |
| 359 | 0.382 | 0.352 | 0.331 | 0.32 | 0.315 | 0.305 | 0.31 | 0.305 | 0.325 | 0.333 |
| 360 | 0.384 | 0.354 | 0.332 | 0.321 | 0.316 | 0.306 | 0.311 | 0.306 | 0.326 | 0.334 |
| 361 | 0.386 | 0.355 | 0.334 | 0.322 | 0.317 | 0.308 | 0.313 | 0.308 | 0.328 | 0.336 |
| 362 | 0.388 | 0.356 | 0.335 | 0.323 | 0.318 | 0.309 | 0.313 | 0.309 | 0.329 | 0.337 |
| 363 | 0.39 | 0.358 | 0.337 | 0.325 | 0.32 | 0.311 | 0.315 | 0.31 | 0.33 | 0.338 |
| 364 | 0.393 | 0.36 | 0.338 | 0.326 | 0.321 | 0.312 | 0.316 | 0.311 | 0.331 | 0.339 |
| 365 | 0.395 | 0.361 | 0.339 | 0.327 | 0.322 | 0.312 | 0.317 | 0.312 | 0.332 | 0.34 |
| 366 | 0.397 | 0.363 | 0.341 | 0.329 | 0.324 | 0.314 | 0.318 | 0.313 | 0.333 | 0.341 |
| 367 | 0.4 | 0.365 | 0.342 | 0.33 | 0.324 | 0.314 | 0.319 | 0.314 | 0.334 | 0.342 |
| 368 | 0.402 | 0.367 | 0.344 | 0.331 | 0.325 | 0.315 | 0.32 | 0.315 | 0.335 | 0.343 |
| 369 | 0.404 | 0.368 | 0.346 | 0.333 | 0.326 | 0.316 | 0.32 | 0.316 | 0.336 | 0.343 |
| 370 | 0.407 | 0.371 | 0.347 | 0.334 | 0.328 | 0.317 | 0.321 | 0.316 | 0.336 | 0.344 |
| 371 | 0.409 | 0.372 | 0.348 | 0.335 | 0.329 | 0.318 | 0.322 | 0.317 | 0.337 | 0.345 |
| 372 | 0.412 | 0.374 | 0.351 | 0.337 | 0.33 | 0.319 | 0.323 | 0.318 | 0.338 | 0.346 |
| 373 | 0.413 | 0.376 | 0.352 | 0.338 | 0.331 | 0.32 | 0.324 | 0.319 | 0.339 | 0.346 |
| 374 | 0.417 | 0.377 | 0.353 | 0.339 | 0.332 | 0.321 | 0.325 | 0.32 | 0.339 | 0.347 |
| 375 | 0.42 | 0.381 | 0.357 | 0.342 | 0.336 | 0.323 | 0.327 | 0.322 | 0.341 | 0.349 |
| 376 | 0.422 | 0.383 | 0.358 | 0.343 | 0.337 | 0.324 | 0.327 | 0.323 | 0.342 | 0.35 |
| 377 | 0.424 | 0.385 | 0.36 | 0.345 | 0.338 | 0.324 | 0.328 | 0.323 | 0.343 | 0.35 |
| 378 | 0.427 | 0.387 | 0.362 | 0.346 | 0.339 | 0.326 | 0.329 | 0.324 | 0.344 | 0.351 |
| 379 | 0.429 | 0.389 | 0.363 | 0.348 | 0.34 | 0.327 | 0.33 | 0.325 | 0.345 | 0.352 |
| 380 | 0.433 | 0.391 | 0.365 | 0.349 | 0.341 | 0.328 | 0.331 | 0.326 | 0.345 | 0.352 |
| 381 | 0.435 | 0.394 | 0.367 | 0.351 | 0.343 | 0.329 | 0.332 | 0.328 | 0.347 | 0.354 |
| 382 | 0.438 | 0.396 | 0.369 | 0.353 | 0.344 | 0.331 | 0.333 | 0.328 | 0.347 | 0.354 |
| 383 | 0.443 | 0.399 | 0.372 | 0.355 | 0.346 | 0.332 | 0.335 | 0.33 | 0.348 | 0.355 |
| 384 | 0.447 | 0.402 | 0.374 | 0.357 | 0.348 | 0.333 | 0.335 | 0.331 | 0.349 | 0.357 |
| 385 | 0.454 | 0.406 | 0.378 | 0.36 | 0.35 | 0.335 | 0.337 | 0.332 | 0.351 | 0.358 |
| 386 | 0.459 | 0.41 | 0.381 | 0.362 | 0.352 | 0.337 | 0.338 | 0.333 | 0.352 | 0.359 |
| 387 | 0.468 | 0.416 | 0.386 | 0.366 | 0.354 | 0.339 | 0.34 | 0.335 | 0.353 | 0.36 |
| 388 | 0.475 | 0.421 | 0.389 | 0.369 | 0.357 | 0.34 | 0.341 | 0.335 | 0.354 | 0.36 |
| 389 | 0.485 | 0.428 | 0.395 | 0.373 | 0.36 | 0.343 | 0.343 | 0.338 | 0.356 | 0.362 |
| 390 | 0.497 | 0.437 | 0.402 | 0.379 | 0.364 | 0.346 | 0.345 | 0.339 | 0.357 | 0.363 |
| 391 | 0.51 | 0.445 | 0.409 | 0.384 | 0.367 | 0.349 | 0.347 | 0.341 | 0.359 | 0.364 |
| 392 | 0.528 | 0.457 | 0.418 | 0.391 | 0.373 | 0.352 | 0.35 | 0.343 | 0.36 | 0.365 |
| 393 | 0.544 | 0.469 | 0.428 | 0.398 | 0.378 | 0.356 | 0.353 | 0.345 | 0.362 | 0.367 |
| 394 | 0.566 | 0.484 | 0.439 | 0.407 | 0.383 | 0.36 | 0.355 | 0.347 | 0.364 | 0.369 |
| 395 | 0.586 | 0.497 | 0.45 | 0.415 | 0.389 | 0.364 | 0.359 | 0.35 | 0.366 | 0.371 |
| 396 | 0.61 | 0.514 | 0.463 | 0.425 | 0.396 | 0.369 | 0.362 | 0.352 | 0.369 | 0.372 |
| 397 | 0.632 | 0.529 | 0.475 | 0.434 | 0.403 | 0.374 | 0.366 | 0.355 | 0.371 | 0.374 |
| 398 | 0.659 | 0.548 | 0.491 | 0.446 | 0.411 | 0.38 | 0.37 | 0.358 | 0.374 | 0.377 |
| 399 | 0.686 | 0.566 | 0.505 | 0.457 | 0.42 | 0.386 | 0.374 | 0.362 | 0.377 | 0.379 |
| 400 | 0.711 | 0.584 | 0.519 | 0.469 | 0.427 | 0.392 | 0.379 | 0.365 | 0.38 | 0.382 |
| 401 | 0.741 | 0.604 | 0.536 | 0.482 | 0.437 | 0.399 | 0.384 | 0.37 | 0.384 | 0.385 |
| 402 | 0.773 | 0.626 | 0.554 | 0.496 | 0.447 | 0.407 | 0.39 | 0.375 | 0.388 | 0.388 |
| 403 | 0.81 | 0.652 | 0.575 | 0.513 | 0.459 | 0.417 | 0.397 | 0.38 | 0.394 | 0.393 |
| 404 | 0.843 | 0.675 | 0.594 | 0.528 | 0.47 | 0.425 | 0.404 | 0.385 | 0.399 | 0.397 |
| 405 | 0.886 | 0.704 | 0.618 | 0.546 | 0.485 | 0.436 | 0.412 | 0.392 | 0.405 | 0.402 |
| 406 | 0.926 | 0.733 | 0.641 | 0.565 | 0.498 | 0.446 | 0.42 | 0.399 | 0.411 | 0.408 |
| 407 | 0.977 | 0.767 | 0.67 | 0.588 | 0.515 | 0.459 | 0.43 | 0.407 | 0.419 | 0.414 |
| 408 | 1.034 | 0.805 | 0.702 | 0.613 | 0.534 | 0.474 | 0.441 | 0.416 | 0.427 | 0.421 |
| 409 | 1.099 | 0.85 | 0.738 | 0.642 | 0.555 | 0.491 | 0.455 | 0.427 | 0.437 | 0.43 |
| 410 | 1.163 | 0.893 | 0.774 | 0.672 | 0.577 | 0.507 | 0.467 | 0.437 | 0.447 | 0.438 |
| 411 | 1.241 | 0.945 | 0.818 | 0.707 | 0.602 | 0.527 | 0.482 | 0.45 | 0.458 | 0.448 |
| 412 | 1.3 | 0.985 | 0.852 | 0.734 | 0.622 | 0.543 | 0.494 | 0.46 | 0.468 | 0.456 |
| 413 | 1.386 | 1.042 | 0.901 | 0.774 | 0.653 | 0.566 | 0.512 | 0.474 | 0.481 | 0.468 |
| 414 | 1.462 | 1.094 | 0.946 | 0.812 | 0.68 | 0.588 | 0.529 | 0.489 | 0.494 | 0.48 |
| 415 | 1.536 | 1.143 | 0.991 | 0.849 | 0.708 | 0.61 | 0.546 | 0.503 | 0.508 | 0.491 |
| 416 | 1.605 | 1.193 | 1.037 | 0.888 | 0.738 | 0.635 | 0.566 | 0.52 | 0.524 | 0.505 |
| 417 | 1.657 | 1.231 | 1.074 | 0.921 | 0.764 | 0.656 | 0.584 | 0.535 | 0.538 | 0.518 |
| 418 | 1.718 | 1.269 | 1.111 | 0.953 | 0.79 | 0.679 | 0.602 | 0.552 | 0.554 | 0.532 |
| 419 | 1.757 | 1.303 | 1.147 | 0.986 | 0.818 | 0.704 | 0.624 | 0.572 | 0.573 | 0.55 |
| 420 | 1.787 | 1.327 | 1.168 | 1.009 | 0.838 | 0.724 | 0.641 | 0.587 | 0.588 | 0.564 |
| 421 | 1.804 | 1.339 | 1.186 | 1.027 | 0.857 | 0.744 | 0.661 | 0.606 | 0.607 | 0.581 |
| 422 | 1.808 | 1.345 | 1.194 | 1.04 | 0.871 | 0.761 | 0.677 | 0.622 | 0.624 | 0.597 |
| 423 | 1.799 | 1.344 | 1.197 | 1.047 | 0.885 | 0.778 | 0.696 | 0.641 | 0.643 | 0.616 |
| 424 | 1.783 | 1.338 | 1.194 | 1.049 | 0.891 | 0.79 | 0.71 | 0.656 | 0.658 | 0.631 |
| 425 | 1.755 | 1.322 | 1.183 | 1.047 | 0.899 | 0.804 | 0.728 | 0.676 | 0.68 | 0.653 |
| 426 | 1.722 | 1.302 | 1.166 | 1.04 | 0.901 | 0.815 | 0.743 | 0.694 | 0.698 | 0.671 |
| 427 | 1.673 | 1.271 | 1.143 | 1.027 | 0.902 | 0.826 | 0.76 | 0.714 | 0.72 | 0.695 |
| 428 | 1.613 | 1.235 | 1.116 | 1.013 | 0.901 | 0.836 | 0.776 | 0.734 | 0.741 | 0.717 |
| 429 | 1.544 | 1.194 | 1.083 | 0.995 | 0.898 | 0.846 | 0.793 | 0.756 | 0.766 | 0.743 |
| 430 | 1.47 | 1.147 | 1.049 | 0.975 | 0.894 | 0.854 | 0.81 | 0.777 | 0.789 | 0.768 |
| 431 | 1.387 | 1.099 | 1.013 | 0.953 | 0.89 | 0.862 | 0.826 | 0.799 | 0.814 | 0.795 |
| 432 | 1.3 | 1.046 | 0.974 | 0.93 | 0.884 | 0.87 | 0.843 | 0.822 | 0.838 | 0.823 |
| 433 | 1.211 | 0.993 | 0.935 | 0.906 | 0.878 | 0.877 | 0.859 | 0.844 | 0.863 | 0.85 |
| 434 | 1.12 | 0.937 | 0.894 | 0.883 | 0.872 | 0.884 | 0.875 | 0.867 | 0.889 | 0.88 |
| 435 | 1.033 | 0.883 | 0.855 | 0.859 | 0.865 | 0.89 | 0.89 | 0.888 | 0.913 | 0.908 |
| 436 | 0.953 | 0.834 | 0.818 | 0.837 | 0.858 | 0.894 | 0.903 | 0.907 | 0.935 | 0.933 |
| 437 | 0.878 | 0.785 | 0.783 | 0.815 | 0.85 | 0.898 | 0.914 | 0.924 | 0.955 | 0.956 |
| 438 | 0.799 | 0.735 | 0.746 | 0.791 | 0.841 | 0.899 | 0.925 | 0.942 | 0.975 | 0.98 |
| 439 | 0.734 | 0.691 | 0.713 | 0.77 | 0.831 | 0.899 | 0.932 | 0.955 | 0.99 | 1 |
| 440 | 0.665 | 0.645 | 0.678 | 0.746 | 0.819 | 0.896 | 0.937 | 0.966 | 1.005 | 1.019 |
| 441 | 0.614 | 0.61 | 0.65 | 0.726 | 0.808 | 0.891 | 0.938 | 0.972 | 1.012 | 1.03 |
| 442 | 0.557 | 0.57 | 0.619 | 0.702 | 0.793 | 0.883 | 0.936 | 0.975 | 1.018 | 1.04 |
| 443 | 0.506 | 0.533 | 0.588 | 0.678 | 0.776 | 0.871 | 0.929 | 0.973 | 1.019 | 1.045 |
| 444 | 0.468 | 0.505 | 0.565 | 0.658 | 0.761 | 0.858 | 0.921 | 0.968 | 1.014 | 1.043 |
| 445 | 0.432 | 0.477 | 0.54 | 0.636 | 0.742 | 0.842 | 0.908 | 0.958 | 1.006 | 1.039 |
| 446 | 0.399 | 0.452 | 0.516 | 0.614 | 0.723 | 0.824 | 0.892 | 0.944 | 0.993 | 1.029 |
| 447 | 0.37 | 0.427 | 0.493 | 0.591 | 0.701 | 0.802 | 0.872 | 0.925 | 0.977 | 1.014 |
| 448 | 0.347 | 0.408 | 0.474 | 0.572 | 0.682 | 0.781 | 0.853 | 0.907 | 0.958 | 0.998 |
| 449 | 0.322 | 0.385 | 0.451 | 0.547 | 0.656 | 0.754 | 0.825 | 0.879 | 0.932 | 0.973 |
| 450 | 0.304 | 0.369 | 0.434 | 0.528 | 0.636 | 0.73 | 0.801 | 0.856 | 0.907 | 0.95 |
| 451 | 0.285 | 0.35 | 0.413 | 0.504 | 0.609 | 0.7 | 0.77 | 0.823 | 0.874 | 0.919 |
| 452 | 0.268 | 0.333 | 0.394 | 0.481 | 0.582 | 0.668 | 0.737 | 0.789 | 0.839 | 0.884 |
| 453 | 0.255 | 0.319 | 0.378 | 0.461 | 0.558 | 0.64 | 0.706 | 0.756 | 0.806 | 0.851 |
| 454 | 0.244 | 0.307 | 0.362 | 0.441 | 0.534 | 0.611 | 0.676 | 0.724 | 0.772 | 0.815 |
| 455 | 0.234 | 0.296 | 0.348 | 0.422 | 0.511 | 0.583 | 0.645 | 0.691 | 0.737 | 0.781 |
| 456 | 0.224 | 0.284 | 0.333 | 0.402 | 0.485 | 0.552 | 0.61 | 0.654 | 0.698 | 0.74 |
| 457 | 0.217 | 0.274 | 0.32 | 0.385 | 0.463 | 0.524 | 0.58 | 0.621 | 0.664 | 0.705 |
| 458 | 0.209 | 0.264 | 0.307 | 0.366 | 0.439 | 0.495 | 0.548 | 0.585 | 0.627 | 0.665 |
| 459 | 0.203 | 0.255 | 0.294 | 0.349 | 0.416 | 0.467 | 0.515 | 0.55 | 0.589 | 0.626 |
| 460 | 0.198 | 0.249 | 0.285 | 0.335 | 0.399 | 0.445 | 0.491 | 0.523 | 0.56 | 0.595 |
| 461 | 0.193 | 0.242 | 0.274 | 0.321 | 0.38 | 0.421 | 0.464 | 0.493 | 0.529 | 0.562 |
| 462 | 0.19 | 0.236 | 0.265 | 0.307 | 0.362 | 0.399 | 0.438 | 0.465 | 0.499 | 0.53 |
| 463 | 0.186 | 0.23 | 0.257 | 0.295 | 0.345 | 0.377 | 0.414 | 0.439 | 0.471 | 0.499 |
| 464 | 0.183 | 0.224 | 0.248 | 0.282 | 0.327 | 0.355 | 0.389 | 0.41 | 0.441 | 0.467 |
| 465 | 0.18 | 0.22 | 0.241 | 0.272 | 0.314 | 0.338 | 0.369 | 0.389 | 0.417 | 0.442 |
| 466 | 0.178 | 0.215 | 0.234 | 0.262 | 0.299 | 0.32 | 0.348 | 0.365 | 0.393 | 0.415 |
| 467 | 0.176 | 0.212 | 0.229 | 0.254 | 0.289 | 0.306 | 0.333 | 0.348 | 0.374 | 0.395 |
| 468 | 0.174 | 0.209 | 0.224 | 0.245 | 0.277 | 0.292 | 0.317 | 0.331 | 0.355 | 0.374 |
| 469 | 0.173 | 0.205 | 0.219 | 0.237 | 0.267 | 0.278 | 0.301 | 0.313 | 0.336 | 0.354 |
| 470 | 0.172 | 0.203 | 0.215 | 0.232 | 0.259 | 0.268 | 0.289 | 0.3 | 0.322 | 0.339 |
| 471 | 0.171 | 0.201 | 0.21 | 0.225 | 0.25 | 0.257 | 0.276 | 0.286 | 0.307 | 0.322 |
| 472 | 0.17 | 0.199 | 0.208 | 0.221 | 0.243 | 0.249 | 0.266 | 0.275 | 0.295 | 0.31 |
| 473 | 0.17 | 0.197 | 0.205 | 0.216 | 0.237 | 0.241 | 0.257 | 0.265 | 0.284 | 0.298 |
| 474 | 0.17 | 0.196 | 0.202 | 0.212 | 0.231 | 0.233 | 0.248 | 0.254 | 0.273 | 0.286 |
| 475 | 0.169 | 0.195 | 0.2 | 0.209 | 0.226 | 0.227 | 0.241 | 0.246 | 0.265 | 0.277 |
| 476 | 0.169 | 0.194 | 0.198 | 0.205 | 0.221 | 0.22 | 0.234 | 0.238 | 0.256 | 0.267 |
| 477 | 0.169 | 0.193 | 0.197 | 0.203 | 0.218 | 0.216 | 0.228 | 0.232 | 0.25 | 0.26 |
| 478 | 0.17 | 0.193 | 0.195 | 0.2 | 0.214 | 0.211 | 0.223 | 0.226 | 0.243 | 0.253 |
| 479 | 0.17 | 0.192 | 0.194 | 0.198 | 0.211 | 0.207 | 0.219 | 0.222 | 0.238 | 0.248 |
| 480 | 0.17 | 0.192 | 0.194 | 0.197 | 0.209 | 0.204 | 0.215 | 0.217 | 0.233 | 0.243 |
| 481 | 0.171 | 0.192 | 0.193 | 0.195 | 0.206 | 0.201 | 0.212 | 0.214 | 0.229 | 0.238 |
| 482 | 0.171 | 0.192 | 0.192 | 0.194 | 0.205 | 0.199 | 0.208 | 0.21 | 0.225 | 0.234 |
| 483 | 0.172 | 0.192 | 0.192 | 0.193 | 0.203 | 0.197 | 0.206 | 0.207 | 0.222 | 0.231 |
| 484 | 0.173 | 0.192 | 0.191 | 0.192 | 0.201 | 0.195 | 0.203 | 0.205 | 0.219 | 0.228 |
| 485 | 0.173 | 0.192 | 0.191 | 0.191 | 0.2 | 0.193 | 0.202 | 0.203 | 0.217 | 0.225 |
| 486 | 0.174 | 0.192 | 0.191 | 0.191 | 0.199 | 0.192 | 0.2 | 0.201 | 0.215 | 0.223 |
| 487 | 0.175 | 0.192 | 0.191 | 0.191 | 0.199 | 0.19 | 0.199 | 0.199 | 0.213 | 0.221 |
| 488 | 0.175 | 0.193 | 0.191 | 0.19 | 0.198 | 0.19 | 0.198 | 0.198 | 0.212 | 0.219 |
| 489 | 0.176 | 0.193 | 0.191 | 0.19 | 0.198 | 0.189 | 0.197 | 0.197 | 0.211 | 0.218 |
| 490 | 0.177 | 0.194 | 0.191 | 0.19 | 0.197 | 0.188 | 0.196 | 0.196 | 0.209 | 0.217 |
| 491 | 0.178 | 0.195 | 0.192 | 0.19 | 0.197 | 0.188 | 0.195 | 0.195 | 0.209 | 0.216 |
| 492 | 0.179 | 0.195 | 0.192 | 0.191 | 0.197 | 0.187 | 0.195 | 0.194 | 0.208 | 0.215 |
| 493 | 0.181 | 0.196 | 0.193 | 0.191 | 0.197 | 0.187 | 0.194 | 0.194 | 0.208 | 0.214 |
| 494 | 0.182 | 0.197 | 0.194 | 0.191 | 0.197 | 0.187 | 0.194 | 0.194 | 0.207 | 0.214 |
| 495 | 0.184 | 0.198 | 0.194 | 0.192 | 0.197 | 0.187 | 0.194 | 0.194 | 0.207 | 0.214 |
| 496 | 0.186 | 0.199 | 0.195 | 0.193 | 0.198 | 0.188 | 0.194 | 0.194 | 0.207 | 0.214 |
| 497 | 0.187 | 0.2 | 0.196 | 0.193 | 0.198 | 0.188 | 0.194 | 0.194 | 0.207 | 0.214 |
| 498 | 0.189 | 0.202 | 0.198 | 0.194 | 0.199 | 0.188 | 0.195 | 0.194 | 0.207 | 0.214 |
| 499 | 0.192 | 0.204 | 0.199 | 0.195 | 0.2 | 0.189 | 0.195 | 0.194 | 0.207 | 0.214 |
